# Distribution of Virulence Factors and Resistance Determinants in Three Genotypes of *Staphylococcus argenteus* Clinical Isolates in Japan

**DOI:** 10.3390/pathogens10020163

**Published:** 2021-02-03

**Authors:** Meiji Soe Aung, Noriko Urushibara, Mitsuyo Kawaguchiya, Mina Hirose, Miyo Ike, Masahiko Ito, Nobumichi Kobayashi

**Affiliations:** 1 Department of Hygiene, Sapporo Medical University School of Medicine, Hokkaido, Sapporo 060-8556, Japan; noriko-u@sapmed.ac.jp (N.U.); kawaguchiya@sapmed.ac.jp (M.K.); nkobayas@sapmed.ac.jp (N.K.); 2 Division of Pediatric Dentistry, Department of Oral Growth and Development, School of Dentistry, Health Sciences University of Hokkaido, Ishikari-Tobetsu 061-0293, Japan; minaniwa@hoku-iryo-u.ac.jp; 3 Sapporo Clinical Laboratory, Incorporated, Hokkaido, Sapporo 060-0005, Japan; saturin-saikin@saturin.co.jp (M.I.); m-ito@saturin.co.jp (M.I.)

**Keywords:** *Staphylococcus argenteus*, ST, virulence factors, enterotoxin, resistance gene, Japan

## Abstract

*Staphylococcus argenteus*, a novel staphylococcal species independent of *S. aureus*, causes a wide spectrum of infectious diseases. As detection of this species from humans and animals has been increasingly reported worldwide, its growing virulence and drug resistance via external genetic determinants has become concerning. In this study, the prevalence and genetic characteristics of virulence factors and drug resistance determinants were investigated for 82 *S. argenteus* clinical isolates in Hokkaido, Japan, for a one-year period starting in August 2019. These *S. argenteus* isolates corresponded to 0.66% of the total number of *S. aureus* isolates collected in the same period. The most prevalent genotype was sequence type (ST) 2250 and staphylocoagulase (*coa*) genotype XId (45.1%, *n* = 37), followed by ST1223-*coa* XV (30.5%, *n* = 25) and ST2198-*coa* XIV (24.4%, *n* = 20). Panton-Valentine leukocidin genes (*lukS-PV-lukF-PV*) were identified in a single ST2250 isolate. Only ST1223 isolates had the enterotoxin gene cluster (*egc-2*), *seb*, and *selw* (detection rate; 100%, 60%, and 84%, respectively), while *sec*, *sey*, *sel26-sel27*, *tst-1* were only detected in ST2250 isolates (detection rate; 10.8%, 100%, 67.6%, and 10.8%, respectively). ST2198 isolates harbored *selx* at a significantly higher rate (60%) than isolates of other STs. Although most of *S. argenteus* isolates were susceptible to antimicrobials examined, ST2198 showed higher resistance rates to penicillin, macrolides, and aminoglycosides than other STs, and it harbored various resistance genes such as *blaZ*, *erm(C)*, *msr(A)*, *lnuA*, and *aac(6′)-Ie-aph(2″)-Ia*. Only one ST2250 isolate possessed SCC*mec*-IVc, showing resistance to oxacillin. *blaZ* was the most prevalent determinant of resistance in the three STs and belonged to two plasmid groups and a chromosomal group, suggesting its diverse origin. *lnu(A)* in ST2198 isolates was assigned to a major cluster with various staphylococcal species. The present study indicates that the prevalence of virulence factors and drug resistance profile/determinants differ depending on the lineage (ST) of *S. argenteus*.

## 1. Introduction

*Staphylococcus argenteus* is a novel coagulase-positive *Staphylococcus* species that is genetically closely related to *Staphylococcus aureus* and classified within the *S. aureus* complex (SAC) together with *S. aureus*, and *S. schweitzeri* [1]. *S. aureus* and *S. argenteus* are widely distributed in human and animals, while *S. schweitzeri* has been identified mostly in animals in Africa [2,3]. *S. argenteus* shows non-pigmented (creamy white) colonies on blood agar due to lack of the *crtOPQMN* operon responsible for production of carotenoid pigment, staphyloxanthin, which is essential for protection against oxidative stress and neutrophil killing [4]. Genetically divergent *S. aureus* of sequence type 75 (ST75) was first described in 2002 [5], and was later assigned to *S. argenteus* belonging to clonal complex 75 (CC75) for clinical isolates found in the Northern Territory of Australia during a period from 2004 to 2005 [6]. Thereafter, various STs and CCs have been identified for *S. argenteus*, including CC1223, CC2198, CC2250, CC2596 and CC2854 [7], and more than 60 STs have been currently assigned under several clonal complexes. ST2250 is considered to be the dominant clone with widespread geographical distribution including Europe [8,9,10,11,12], Southeast Asia [13,14,15,16], China [17], and Japan [18,19,20], while ST1223, the lineage related to CC75, is less prevalent and has been described as the pathogen responsible for outbreaks of food poisoning in Japan [21,22] as well as colonizing isolates in Cambodia and the Amazonian forest [23,24].

In recent years, *S. argenteus* has been increasingly reported worldwide as an emerging pathogen, causing a wide spectrum of infectious diseases such as skin and soft-tissue infections (SSTIs) [6,13], necrotizing fasciitis [4], chronic prosthetic-related infection [25,26,27], bacteremia and invasive endovascular infections [14,19,28] that may have high impact of mortality [29]. It has also been documented as a nasal colonizing isolate in healthy people [30], an environmental isolate in dental clinics [31], and has even been identified in retail foods [32]. The prevalence of *S. argenteus* in presumptive *S. aureus* varies depending on geographical area, specimen type and infection site. A high detection rate (71%) among presumptive community-acquired methicillin-resistant *S. aureus* (CA-MRSA) isolates was documented in Northern Australia [6]. Slightly higher prevalence was reported in Thailand (19% of community-onset isolates causing sepsis [7], 4.1% from invasive infection [33]), Laos (6% in SSTIs) [13] and Myanmar (3.5% in nasal isolates from food handlers [30] and 2.9% in clinical presumptive *S. aureus* isolates [15]). In contrast, low prevalence (<1%) was noted in European countries [9,10,11], eastern China (0.7%) [34], and Japan (1% in blood stream infections [19] and 0.55% in various clinical isolates [20]). Although *S. argenteus* has usually been detected by species-specific PCR or mass spectrometric analysis, the prevalence of *S. argenteus* has probably been underestimated worldwide because sequencing of specific genes is essential for identification of this species.

The majority of *S. argenteus* clinical isolates have been found to be methicillin-sensitive, whereas methicillin-resistant isolates were mostly identified in Australia, belonging to CC75 [2,6,35,36]. In contrast, methicillin-resistant isolates have rarely been detected in other lineages (ST1223, ST2250 and ST2793) in European countries [9,11,37] and Asia [13]. SCC*mec* of methicillin-resistant *S. argenteus* has been classified mostly as type IV, and rarely, as type V [2,9,13,37]. Previous reports have found that *S. argenteus* clinical isolates were susceptible to most of the antimicrobials tested [2,12,30,37], and the beta-lactamase gene *blaZ* has often been associated with them [9,13,15]. However, resistance to non-beta-lactam antimicrobials has been noted recently [29,30,37], furthermore, the occurrence of daptomycin resistance was reported for *S. argenteus* detected in a complex vascular graft infection in the USA [28]. Therefore, acquisition of drug resistance by *S. argenteus* is an issue of growing concern. A genomic study revealed that *S. argenteus* possesses most of the virulence factors of *S. aureus*, suggesting that *S. argenteus* has similar pathogenic potential to that of *S. aureus* [17]. Similarly, many reports have highlighted the pathogenicity and clinical importance of *S. argenteus* as an emerging infectious agent evidenced by the presence of various pathogenic genes [8,16,20,29,30,31].

In Japan, ST1223, ST2198, ST2250 *S. argenteus* has been reported to date: clinical isolates (these three STs) [20], food poisoning outbreaks [21,22] and keratoconjunctivitis due to ST1223 [38], purulent lymphadenitis [18], bacteremia [19] and mycotic aortic aneurysm [39] due to ST2250. Although various infection types are known for *S. argenteus*, information on the epidemiological trends and genetic characteristics of this species is still limited. The current study was conducted to elucidate the prevalence of virulence and drug resistance determinants in individual clones of *S. argenteus* clinical isolates collected for a one-year period in Hokkaido, the most northern of the main islands of Japan.

## 2. Results

### 2.1. Identification and Prevalence of S. argenteus

In a one-year period, we identified a total of eighty-two *S. argenteus* isolates from 82 patients, comprising 81 methicillin-susceptible and one single methicillin-resistant isolate. The isolation ratios of all *S. argenteus* to all *S. aureus*, methicillin-susceptible *S. argenteus* to MSSA, and methicillin-resistant *S. argenteus* to MRSA were 0.0066 (82/12,510), 0.01 (81/8,132), 0.0002 (1/4,378), respectively. This rate represents the very low prevalence of *S. argenteus* in Japan, as reported previously [19,20].

*S. argenteus* isolates were derived from diverse clinical specimens as listed in Table 1. The most common isolation source was the respiratory system (sputum (*n* = 16), nasal discharge (*n* = 11), pharynx (*n* = 6)), followed by stool (*n* = 12), ear discharge (*n* = 10), pus and ear pus (*n* = 9), urine (*n* = 7), and vaginal discharge (*n* = 4). The ratio of outpatients to inpatient was 2.2. The age range of the patients was 0–98 years (average age = 49 years), with a sex ratio (male to female) of 0.64.

### 2.2. Classification of ST and Coagulase Genotype

Among the eighty-two *S. argenteus* isolates, 45.1% (*n* = 37) were classified as ST2250, while 30.5% (*n* = 25) and 24.4% (*n* = 20) of isolates were assigned to ST1223 and ST2198, respectively (Table 1), according to multilocus sequence typing (MLST) of *S. aureus*. Coagulase genotypes, *coa*-XV, *coa*-XIV, and *coa*-XId were identified by sequencing of partial *coa* genes, and were assigned to ST1223, ST2198, and ST2250 isolates, respectively. These identified STs and *coa*-types were the same as those in our previous study [20]. Generally, four to nine *S. argenteus* isolates were identified in each month during the study period, while slightly higher prevalence of all the three STs was observed in October 2019 (Table S1). Three STs were almost uniformly detected in individual months (Table S1), and there was no significant difference in specimens of isolates depending on STs (Table 1) and also virulence factors and resistance determinants as described below. A single methicillin-resistant isolate, which belonged to ST2250 (isolate id: SG99) was detected in June 2020 (Table S2).

### 2.3. Prevalence of Virulence Factors

All the isolates harbored alpha-, beta-, and delta-hemolysin genes (*hla*, *hlb*, *hld*) whereas gamma-hemolysin gene (*hlg)* was identified in 86.6% of total isolates (Table 1 and Table S2). Only an ST2250 isolate from stool (SG38) had Panton-Valentine leukocidin (PVL) genes (*lukS-PV-lukF-PV*), which was assigned to haplotype H1, and its phage type was classified as ϕPVL. None of the isolates had ACME-*arcA*.

Only ST1223 isolates had the enterotoxin gene cluster (*egc-2*; *seg-sei-sem-sen-seo-seu*), *seb*, and *selw* (detection rate; 100%, 60%, and 84%, respectively), while *sec*, *sey*, *sel26-sel27*, *tst-1* were only detected in ST2250 isolates (detection rate; 10.8%, 100%, 67.6%, and 10.8%, respectively). ST2198 isolates harbored *selx* at a significantly higher rate (60%) than isolates of other STs. The nucleotide sequences of *egc-2* determined for five isolates (Table S3) were identical, and showed 98–100% identity to those of *S. argenteus* strains reported previously, while slightly lower identities were evident to *egc-2* components of *S. aureus* strains (92–99%, *seg*, *sei*, *sem*, *seo*, *seu*; 88–90%, *sen*) (Table S3). Although most of the *seb* genes detected in ST1223 isolates were classified as *seb3*, this gene of a single isolate (SG63) was assigned to the new genetic variant *seb6* as well as amino acid variant v4 (Figure S1) after the variant number reported previously [40]. *sea* (*sea2*) and *sec* (*sec3*) were only detected in an ST2198 isolate and four ST2250 isolates, respectively (Table S2). TSST-1 gene (*tst-1*) was found in four ST2250 isolates and its sequence was identical to others reported for *S. aureus* strains (e.g., N315). The prevalence of the components of immune evasion cluster (IEC) (*sak*, *chp* and *scn*) [41] was different depending on STs. Seven isolates of ST2198 harbored IEC, while IEC-A (*sea2*, *sak*, *chp*, *scn*) and IEC-B (*sak*, *chp* and *scn*) were detected in one and six isolates, respectively. Both *sak* and *scn* genes devoid of *chp* (IEC-E) were detected in ST2250 isolates (67.6%), while only *scn* (*sak*, *chp*-negative) was found in 28% of ST1223 isolates. Adhesin genes *ebpS*, *fnbA*, *fnbB* were detected in all the isolates, and *clfB* and *eno* showed high prevalence (80–95%) in all the three STs. Although *sdrC*, *sdrD*, *sdrE* were found in three STs, the prevalence of *sdrC* and *sdrE* was significantly high in ST1223 (72%) and ST2250 (54.1%), respectively.

### 2.4. Antimicrobial Susceptibility and Prevalence of Drug Resistance Genes

Among the 82 isolates studied, only an ST2250 isolate from blood (SG99) was *mecA*-positive and showed resistance to oxacillin and cefoxitin (Table 2 and Table S2), according to the determination of minimum inhibitory concentrations by broth microdilution tests. Sequence analysis of its whole SCC*mec* region, identified its genotype as type IV, subtype c (SCC*mec*-IVc), with highest identity (>99%) to that of MRSA strains TCH60 and NN1 belonging to ST30 [42,43]. Ampicillin resistance was found in 19.5% (16/82) of isolates, all of which harbored *blaZ* gene (Table 2 and Table S2). *blaZ* was detected in ST2198 at a significantly higher rate (60%, 12/20) while it was distributed in all three STs.

Resistance to macrolides and lincosamides was only found in ST2198 isolates. Seven ST2198 isolates showed resistance to erythromycin, clarithromycin, and azithromycin, and harbored one or two genes of *msr(A)*, *lnu(A)*, and *erm(C)*, with *msr(A)* being the most common (5 isolates, 25% of ST2198). Four *lnu(A*)-positive isolates were resistant to lincomycin, and were susceptible to clindamycin. Only an isolate (SG70) had *erm(C)*, together with *lnu(A)*, and showed constitutive resistance to clindamycin, which was confirmed by the D-zone test. Resistance to tetracycline was detected in an ST2250 isolate with *tet(K)*, which was sensitive to doxycycline and minocycline. An ST1223 isolate and five ST2198 isolates that harbored *aac(6′)-Ie-aph(2″)-Ia* were resistant to gentamicin with high MIC levels (≥512 μg/mL), while *aph (3′)-IIIa* was detected in two ST2250 isolates with kanamycin resistance.

### 2.5. Genetic Characterization of Drug Resistance Determinants

The nucleotide sequences of aminoglycoside, macrolide, and tetracycline resistance determinants *aac(6′)-Ie-aph(2″)-Ia*, *aph(3′)-IIIa*, *erm(C)*, *msr(A)*, and *tet(K)* of *S. argenteus* isolates were almost identical (99–100% identity) to those of *S. aureus* and coagulase-negative staphylococcal species (Table S4). *aac(6′)-Ie-aph(2″)-Ia* detected in six isolates was located between IS*256* (IS*256*-flanking pattern A [44]). The *erm(C)* promoter region of *S. argenteus* isolate SG70 lacked a 107-bp sequence containing an ORF of leader peptide (19-amino acids), which is attributable to inducible expression of *erm(C)* [45] (Figure S2). Similar deletion in *erm(C)* regulator regions was identified in many sequences of *S. aureus* plasmid (>70) by BLAST search (data not shown), while *S. argenteus* strain M260_MSHR was found to have an intact leader peptide sequence (Figure S2).

**Figure 1:**
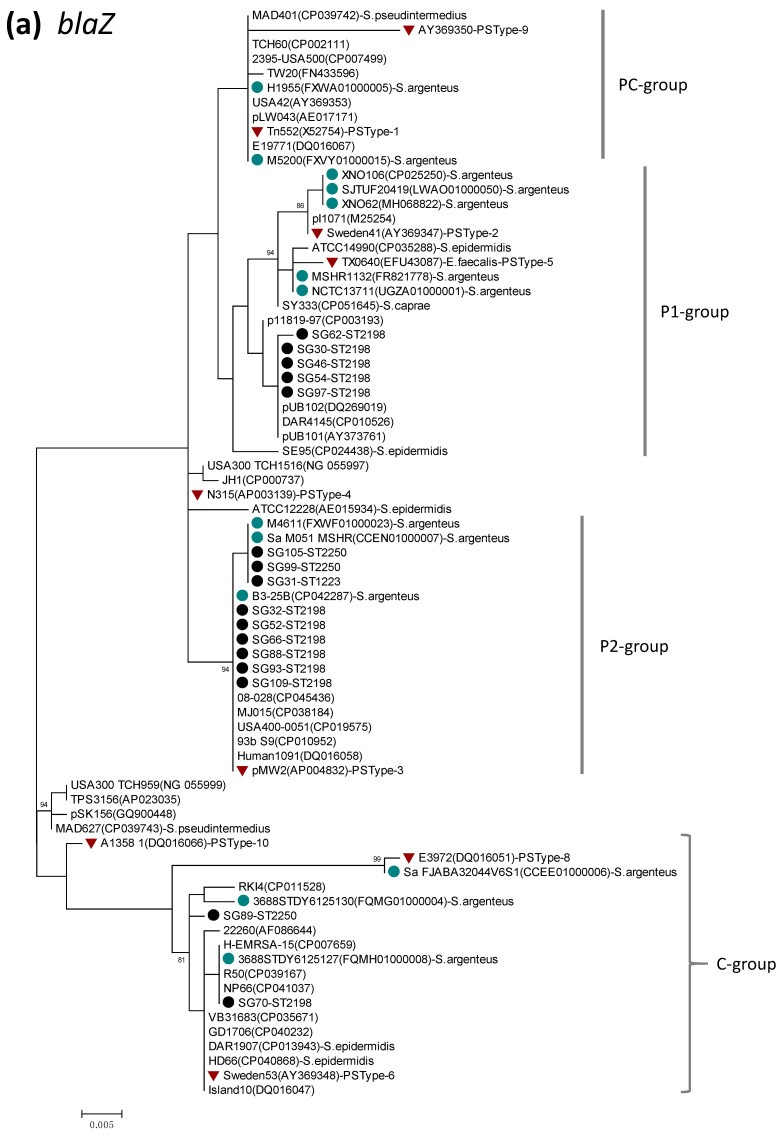

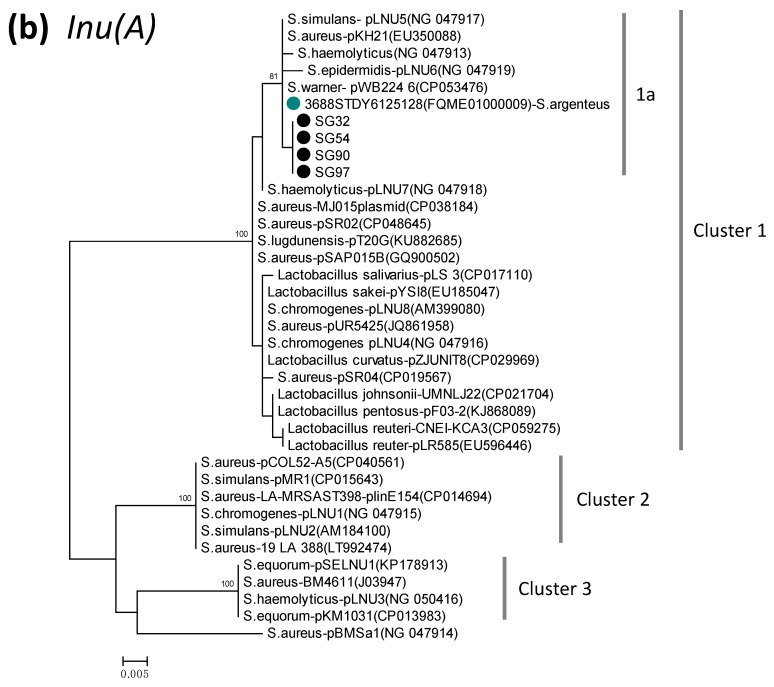
Phylogenetic dendrogram of *blaZ* (**a**) and *lnu(A)* (**b**), constructed by the maximum likelihood method using MEGA X. The tree was statistically supported by bootstrapping with 1000 replicates, and genetic distances were calculated by the Kimura two-parameter model. Variation scale is provided at bottom. Percentage bootstrap support is indicated by values at each node (values <80 are omitted). Closed black and blue circles indicate *S. argenteus* isolates analyzed in the present study and strains available in the GenBank database, respectively. Inverted triangles denote genes of representative BlaZ protein sequence types (PS types) described previously [46], by which P (P1, P2)-, C-, and PC-groups were assigned as shown on the right (a). Cluster numbers 1–3 and subcluster 1a are shown on the right (b). The species name *S. aureus* was omitted (**a**), and GenBank accession numbers are shown in parenthesis followed by strain names.

Clear genetic diversity was revealed for *blaZ* and *lnu(A)*. Based on origin, *blaZ* genes distributed to staphylococcal species have been classified by Olsen and coworkers into the plasmid (P) group, chromosomal (C) group, and intermediate (PC) group [46]. In the present study, phylogenetic analysis of *blaZ* was performed using the sequence data of the current 16 *S. argenteus* isolates, together with those of *S. aureus*, *S. argenteus*, coagulase-negative staphylococcus, and enterococcus (total 59 strains) obtained from the GenBank database. The phylogenetic dendrogram of *blaZ* (Figure 1a) indicated the presence of the three genetic groups, with the P-group being divided into two subgroups (P1, P2). *blaZ* of *S. argenteus* in the present study were mostly classified into the P-group (P1 and P2), while two isolates were assigned to the C-group. *blaZ* sequences of *S. argenteus* retrieved from the GenBank database clustered mostly in the P-group, with some strains being allocated to the C- or PC-groups. *blaZ* from ST2198 was assigned to the P1 group (5 isolates), P2 group (6 isolates) and C group (1 isolate). The sequence identity of *blaZ* between the P1- and P2-groups was 97.8–99.1%, while these groups had 94.2–95.4% identity to the C-group.

A phylogenetic dendrogram of *lnu(A)* from *S. argenteus* and other bacterial species (Figure 1b) indicated the presence of three genetic groups (cluster 1–3). Cluster 1 was a major group including various species (98.6–99.8% identity within this cluster), and showed 92–93% identity to cluster 2 and 3. Four ST2198 *S. argenteus* isolates were assigned to cluster 1, and further to subcluster 1a along with only one sequence of *S. argenteus* strain available in GenBank (strain 3688STDY6125128, Thailand), *S. aureus*, and coagulase-negative staphylococcus. *lnu(A)* of *S. aureus* was dispersed in the three clusters.

Although *S. argenteus* isolates in the present study were susceptible to levofloxacin, four genes encoding targets of fluoroquinolone (*gyrA*, *gyrB*, *grlA*, *grlB*) were analyzed for their diversity from *S. aureus*. The sequence of these genes were determined for five isolates belonging to three STs, and were analyzed with the sequence data of *S. argenteus* strains, MSHR1132 and XNO106. Within the *S. argenteus* strains, the nucleotide sequence identity of the four genes was 99.6–100%, while *S. argenteus* showed 93.4% identity of *gyrA*/*gyrB* and 87.7% identity of *grlA*/*grlB* to those of *S. aureus* strains. Despite such diversity, deduced amino acid sequences of the quinolone-resistance determining region in the four proteins (GyrA, GyrB, ParC, ParE) were identical in *S. argenteus* and fluoroquinolone-susceptible *S. aureus* strains (N315, NCTC8325) (Figure S3) [47,48,49].

## 3. Discussion

In the present study, the prevalence and genetic characteristics of *S. argenteus* clinical isolates were investigated for a one-year period in northern Japan. The ratio of *S. argenteus* to all the *S. aureus* clinical isolates was 0.0066, which was comparable to that in our preceding study (0.0055, March–June, 2019) [20]. Among the three STs identified, ST2250 accounted for approximately half of the isolates, while the remaining was split into ST1223 (30.5%) and ST2189 (24.4%). A similar proportion of the three STs was also found previously at the same study site [20]. Moreover, *S. argenteus* of the three STs was isolated almost equally in each month throughout the year. In Japan, ST1223 and ST2250 *S. argenteus* have been reported in various types of infectious diseases, with ST1223 being mainly detected in food poisoning outbreaks [18,19,21,22,38,39]. While the predominance of ST2250 has been reported worldwide, ST1223 and ST2198 were less frequently detected in Asian countries [7,13,14,15,16,17,33]. In a recent study in Taiwan of blood isolates, ST1223 and ST2198 accounted for 11% and 2%, respectively [16]. Thus, our present and previous study [20] may indicate a distinct epidemiological feature of *S. argenteus* clones in northern Japan, i.e., relatively higher prevalence of ST1223 and ST2189. Higher mortality risk was reported for bacteremia due to *S. argenteus* compared to MSSA in Taiwan [29]. However, in our present study, most isolates were derived from specimens other than blood, and information about patients’ mortality rate was not available. Thus, the clinical impact of *S. argenteus* infection was not able to be evaluated.

It was notable in the present study that distribution of staphylococcal enterotoxin (SE)-like genes was distinctive depending on the three STs; *seb*, *egc-2*, and *selw* in ST1223, *sey* and *sel26-sel27* in ST2250, and the dominance of *selx* in ST2198. Similar findings regarding the correlation of ST and toxin genes have been reported previously for *S. argenteus* isolates in Japan [19,20,21,22], Taiwan [16] and Myanmar [15]. Our study on the prevalence of newer SE(-like) genes in CA-MRSA revealed lower detection rates of *selz* (5.6%) and *sel26-sel27* (0%) [50]. In contrast, *S. argenteus* isolates in the current study showed a higher prevalence (*selz*, 23.2%; *sel26-sel27*, 30.5%), indicating that these SE-like genes are more specifically distributed to *S. argenteus*. Differences in the prevalence of virulence factors may be related to pathogenicity and the clinical symptoms caused by infection with each ST. For example, the ST1223 strain has been reported as a pathogen of food poisoning, probably due to *seb* and *egc-2* [21,22], and these genes were more commonly detected in ST1223 than other STs in isolates from retail food [32]. PVL genes in ΦPVL were detected in an ST2250 isolate from stool sample in our study, as first report in *S. argenteus* in Japan. Despite their very low prevalence in *S. argenteus*, PVL genes have been detected in ST2250 and ST2277 (related to ST2250) isolates from blood samples (sepsis) in France, in cases with an epidemiological link to Mayotte [8], and in 16% of *S. argenteus* isolates (all ST2250) from sepsis in Thailand [7]. Meanwhile, PVL genes were detected in *S. argenteus* isolates belonging to ST2250 from the nasal cavity of healthy humans in Myanmar [30] and the United Arab Emirates [31]. Although there is still little information, it is possible that the major clone ST2250 may rarely acquire PVL phage in regions where PVL-positive *S. aureus* is commonly distributed (e.g., Southeast Asia [51,52]), and spread locally via the movement of healthy carriers. Further evaluation may be necessary to investigate the clinical impact of PVL-positive *S. argenteus*, because it has been implicated in severe symptoms such as sepsis.

Although *S. argenteus* has been described as having the most virulence genes of *S. aureus* [17], there seems to be a distinct difference in prevalence of virulence factors depending on the lineages of these staphylococcal species. For example, *sek*, *seq* and *speG* were commonly found in PVL-positive ST8-SCC*mec*IVa (USA300 clone), and *sec*, *sel*, *sep*, *tst-1*, *sasL* were widely distributed among PVL-negative ST8-SCC*mec*IVl, while *seb*, *tst-1* and *egc-2* were associated with CC5 (ST5/ST764) MRSA isolates in both community and hospital settings [53,54,55]. In contrast, *sec* and *tst-1* were rarely found, and *sek*, *seq*, *sasL* and *speG* were not identified in *S. argenteus* isolates [20,30].

Methicillin-resistant *S. argenteus* has been primarily identified in Australia as a common cause of community-onset skin infections, accounting for approximately 70% of presumptive MRSA isolates, and belongs to CC75 (ST75, ST1850), having mostly SCC*mec*-IV, rarely SCC*mec*-V [4,5,6,35]. *mecA*-positive *S. argenteus* strains were reported at very low frequency in European countries (UK, Belgium, Sweden) [1,2,9,11,37], revealing ST1223, ST2250, ST2793, and ST3240 (single-locus variant of ST2250) isolates with SCC*mec*-IV. In contrast, in Southeast Asia, methicillin-resistant *S. argenteus* has scarcely been detected despite prevalent areas of *S. argenteus*, following Australia, except for a single isolate of ST2250-SCC*mec*-IV in Lao PDR [13]. Although there had been no reports in Eastern Asia, in our study, methicillin-resistant *S. argenteus* isolate was first identified in Japan, and its genotype was clarified as ST2250/SCC*mec*-IV, the same trait as those of isolates in Belgium and Lao PDR [9,13]. Type IV SCC*mec* is known as a major type of community-acquired MRSA and is distributed to various clones including ST1, ST8, ST30, ST59, ST72, and ST80, among which ST30-SCC*mec*-IV was originally widely distributed to Asia, followed by spread to European countries [56,57]. In our study, SCC*mec* of the *S. argenteus* was assigned to subtype c (SCC*mec*-IVc) based on the J1 region of SCC*mec* [58], and the whole SCC*mec* sequence was almost identical to that of strains TCH60 (CC30, USA) [42] and NN1 (ST30, Japan) [43]. Actually, SCC*mec*-IVc was the most commonly found in ST30 isolates [59]. These findings suggested that the methicillin-resistant *S. argenteus* in our study in Japan may be close related to ST30 MRSA, which is locally distributed. In contrast, the presence of SCC*mec*-IVa was described for CC75 (ST1850) strain MSHR1132 in Australia [4], suggesting that the origin of SCC*mec* in ST1850 might be different from that of ST2250 *S. argenteus*.

Drug resistance rates in *S. argenteus* have been described as lower than those in *S. aureus*, while penicillin resistant isolates due to *blaZ* are common [2,7,12,13,15,29]. Although similar finding was observed in the present study, it was noted that ST2198 isolates exhibited significantly higher resistance rates than other STs to penicillin, macrolide-lincosamide, and aminoglycosides, associated with responsible resistance genes that have almost identical sequences to those known for *S. aureus* and other staphylococci. It is suggested that the higher prevalence of drug resistance is ascribable to either clonal spread of resistant strains among ST2198, or the unknown potential of ST2198 to acquire drug resistance genes via plasmid from other staphylococcal species.

Detection of *blaZ* was described in different clones (STs) of *S. argenteus* [12,13,14]. Phylogenetic analysis of *blaZ* in the present study indicated that *blaZ* in *S. argenteus* belongs mostly to the plasmid group, while some were assigned to the chromosomal group, irrespective of ST. Furthermore, *blaZ* of the plasmid group was discriminated into two distinct groups. These findings may suggest that *blaZ* of *S. argenteus* is of multiple origin, with most of them being derived from different plasmids.

Lincosamide resistance of *S. argenteus* in our study was considered to be mediated by *lnu(A)*, which encodes lincosamide nucleotidyltransferase [60]. This gene has been rarely reported in *S. argenteus*, but was identified in four ST2198 isolates in the present study and was phylogenetically clustered with only one available *S. argenteus* gene in the GenBank database (the strain in Thailand), and also *S. aureus* and coagulase-negative staphylococci in a major cluster. Since *lnu(A)* in staphylococci is located in plasmid [60], it was suggested that plasmid carrying the major type of *lnu(A)* might have been transmitted to *S. argenteus* from other staphylococcal species. The erythromycin ribosomal methylase gene *erm(C)* is most widely distributed in staphylococcus via plasmid and is responsible for macrolide and lincosamide resistance [61], however, it has rarely been found in *S. argenteus*, except in strain M260_MSHR in Australia [1] and SG70 in the present study. Expression of *erm(C)* is inducible by the presence of macrolides when *erm(C)* gene is preceded by a short leader cistron (*ermCL*; ORF of 19 amino acid-peptide) [62]. *S. argenteus* strain M260_MSHR has the intact *ermCL* (Figure S2). However, mutations in the regulator region of *erm(C)*, including deletion of *ermCL*, have been revealed to cause constitutive resistance to macrolides and lincosamides [45,62,63]. *S. argenteus* strain SG70 showed constitutive resistance to clindamycin, associated with deletion of 107-bp sequence containing *ermCL* in the *erm(C)* promoter region. Although this type of deletion mutation in *erm(C)* promoter has not been described in experimentally selected resistant clones, it might have already been widely distributed to *S. aureus* (e.g., USA300_FPR3757, Figure S2) as evidenced by the BLAST search in the present study.

In the present study, low rates of resistance were confirmed to tetracyclines and aminoglycosides due to efflux protein defined by *tet(K)* and aminoglycoside modifying enzymes encoded by *aac(6′)-Ie-aph(2″)-Ia* and *aph(3′)-IIIa*, respectively. Although similar findings were reported previously [7,20], no such resistance was observed for clinical isolates in other studies [7,12,19]. However, higher prevalence of tetracycline resistance [29] and *tet(L)*/*aph(3′)-III* [14] was described for blood isolates in Taiwan and Thailand, respectively. Notably, relatively high resistance rates to multiple antimicrobials including tetracycline/aminoglycoside were documented for *S. argenteus* from retail food (mostly meat and fish) [32]. Thus, the prevalence of tetracycline/aminoglycoside resistance in clinical *S. argenteus* of isolates should be carefully monitored, and attention should be given to animals and foodstuff as potential sources of drug resistance genes or resistant clones to humans. Fluoroquinolone resistance in *S. argenteus* clinical isolates has hardly been detected, while ciprofloxacin resistance was reported in isolates from retail food (meat) at very low rates [32]. In our present study, deduced QRDR amino acid sequences of DNA gyrase and topoisomerase IV subunits of fluoroquinolone-susceptible *S. argenteus* were confirmed to be identical to those of fluoroquinolone-susceptible *S. aureus*, despite evident genetic diversity (88–93% identity) of these proteins in these two species. This finding suggests that the target sites of fluoroquinolone in *S. argenteus* may be identical to those of *S. aureus*, and therefore, fluoroquinolone resistance in *S. argenteus* will probably be caused by mutation in QRDR as described for *S. aureus* [47,48,49].

In conclusion, here we have described the constant prevalence of three STs of *S. argenteus* clinical isolates, the distinctive distribution of virulence factors and drug resistance genes in the three STs based on a one-year surveillance study in northern Japan, and identified PVL-positive and SCC*mec*-IVc-positive isolates. Because the occurrence and potential spread of drug resistance/resistance genes in *S. argenteus* are of concern, further epidemiological study is necessary to monitor the phenotypic and genetic traits of this staphylococcal species.

## 4. Materials and Methods

### 4.1. Bacterial Isolates, Species Identification

*S. argenteus* were isolated from various clinical specimens that were submitted to the Sapporo Clinical Laboratory Inc., Sapporo, Japan, from medical facilities in Hokkaido for one year between August 2019 and July 2020. The clinical specimens were inoculated onto blood agar plates incubated aerobically at 37 °C for 24 h. Gram-positive, coagulase-positive isolates were collected for further study by bacteriological identification. Initial screening of *S. argenteus* was performed by MALDI-TOF mass spectrometry using MALDI Biotyper (BRUKER). Isolates assigned as *S. argenteus* were confirmed genetically, by PCR and sequencing of NRPS and thermostable nuclease gene (*nuc*) using previously reported primers and conditions [17,20]. During the same study period, the number of non-duplicate *S. aureus* isolates (single isolate per patient) was 12,510, which contained 8132 methicillin-susceptible *S. aureus* (MSSA) and 4378 MRSA isolates.

### 4.2. Antimicrobial Susceptibility Testing

Minimum inhibitory concentrations (MICs) within limited ranges were measured by broth microdilution tests against 18 antimicrobial agents (oxacillin, OXA; ampicillin, AMP; cefazolin, CFZ; cefmetazole, CMZ; flomoxef, FMX; imipenem, IPM; gentamicin, GEN; arbekacin, ABK; erythromycin, ERY; clindamycin, CLI; vancomycin, VAN; teicoplanin, TEC; linezolid, LZD; minocycline, MIN; Fosfomycin, FOF; levofloxacin, LVX; cefoxitin, FOX; trimethoprim/sulfamethoxazole, SXT) by using the Dry Plate ‘Eiken’ DP32 (Eiken Chemical, Tokyo, Japan). In addition, MIC was manually measured by broth microdilution for OXA, GEN, ERY, azithromycin (AZM), clarithromycin (CLA), doxycycline (DOX), kanamycin (KAN), lincomycin (LIN), quinupristin-dalfopristin (Q-D), tetracycline (TET), and virginiamycin/pristinamycin (V-G). Resistance or susceptibility was judged according to the breakpoints defined in the Clinical Laboratory Standards Institute (CLSI) guidelines [64] for most of the antimicrobial drugs examined. For fosfomycin and arbekacin, whose break points are not defined by CLSI guidelines, the European Committee on Antimicrobial Susceptibility Testing (EUCAST) breakpoint (FOF, 32 μg/mL, Staphylococcus spp.) [65] and a unique breakpoint (ABK, 4 μg/mL, which is higher than the 2 μg/mL defined by the Japanese Society of Chemotherapy for respiratory infection) [66] were used. For flomoxef, a breakpoint defined by the Japanese Society of Chemotherapy for urinary tract infection (16 μg/mL) [66] was applied.

### 4.3. Genetic Typing, Detection of Virulence Factors and Drug Resistance Genes

For all the isolates, the presence of staphylococcal 16s rRNA, *nuc*, *mecA*, PVL genes and ACME-*arcA* (arginine deiminase gene) was examined by multiplex PCR assay as described by Zhang et al. [67]. Sequence type (ST) was assigned according to multilocus sequence typing (MLST) https://pubmlst.org/ (accessed on 20 December 2020) [68]. Staphylocoagulase genotype (*coa*) was determined by partial sequencing of staphylocoagulase gene (D1, D2, and the central regions) and their highly similar staphylocoagulase sequences were searched by the Basic Local Alignment Search Tool (BLAST: https://blast.ncbi.nlm.nih.gov/Blast.cgi (accessed on 20 December 2020)) as described previously [69].

The presence of 28 staphylococcal enterotoxin (SE) (-like) genes (*sea-see*, *seg-selu*, *selx*, *sely*, *selw*, *selz*, *sel26* and *sel27*), the TSST-1 gene (*tst-1*) and exfoliative toxin genes (*eta*, *etb* and *etd*), leukocidins (*lukDE* and *lukM*), haemolysins (*hla*, *hlb*, *hld* and *hlg*), adhesin genes (*eno*, *cna*, *sdrC*, *sdrD*, *sdrE*, *fib*, *clfA*, *clfB*, *fnbA*, *fnbB*, *icaA*, *icaD*, *ednA*, *ednB*, *bap* and *vWbp*), and modulators of host defense (*sak*, *chp* and *scn*) were analyzed by multiplex or uniplex PCRs [15,20,30,50]. PVL-encoding phage (ϕ108, ϕPVL, ϕSa2958, ϕSa2MW, ϕSLT, ϕTCH60, ϕSa2usa, and ϕSa119) for a PVL-positive isolate was classified by multiplex or uniplex PCR as described previously [51], the full-length *lukS-PV/lukF-PV* sequences were determined by PCR and direct sequencing of PCR products. PVL haplotypes based on single-nucleotide polymorphism profiles were assigned according to the previously reported [51].

Genes conferring resistance to penicillin (*blaZ*), tetracycline (*tet(K)*, *tet(L)*, and *tet(M)*), macrolides-lincosamides-streptogramins (*erm(A)*, *erm(B)*, *erm(C)*, *erm(F)*, *erm(T)*, *erm(X)*, *erm(Y)*, *msr(A)*, *lnu(A)*, *lnu(B)*, *vgaA*, *vgaB*, *vgaD*, *vgbA*, *vgbB*, *vatA*, *vatB*, *vatC*, *vatD*, *vatE*, *vatG*, *IsaA*, *IsaE*, *mefA/E*), aminoglycoside (aminoglycoside modifying enzyme (AME) genes; *aac(6′)-Im*, *aac(6′)-Ie-aph(2″)-Ia*, *ant(3″)-Ia*, *ant(4′)-Ia*, *ant(6)-Ia*, *ant(9)-Ia*, *ant(9)-Ib*, *aph(2″)-Ib*, *aph(2″)-Ic*, *aph(2″)-Id*, and *aph(3′)-IIIa*), linezolid (*optrA*), chloramphenicol (*cfr*) were detected by uniplex or multiplex PCR using the primers previously reported [44,54,70,71] and newly designed in this study (Table S5). For isolates harboring *erm* gene, inducible macrolide-resistance phenotype was confirmed by the D-zone test, in which erythromycin and clindamycin disks are placed in an adjacent position 15 mm apart on Mueller-Hinton agar plate [64].

### 4.4. Sequencing and Phylogenetic Analysis of Drug Resistance Genes and Enterotoxin Genes 

Nucleotide sequences of full-length ORF were determined for *blaZ*, *lnu(A)*, *erm(C)*, *msr(A)*, AME genes, *gyrA/B*, *parC/E* genes by PCR with primers listed in Table S5, followed by Sanger sequencing using the BigDye Terminator v3.1 Cycle Sequencing kit (Applied Biosystems, Foster City, CA, USA) on an automated DNA sequencer (ABI PRISM 3100). Entire ORF regions of individual genes were covered by PCR products. Accuracy of sequence data obtained from DNA sequencer was checked by referring to chromatogram data. Similarly, nucleotide sequences of the whole region of SCC*mec*, enterotoxin genes detected (*sea*, *seb*, *sec*, *egc-2 cluster*, *selx*, *sey*, *selw*, *sel26-sel27*), *tst-1*, and PVL genes were also determined. Phylogenetic dendrograms of drug resistance genes were constructed by the maximum likelihood method using the MEGA X software, together with sequence data of staphylococcal strains available in the GenBank database. The dendrograms were statistically supported by bootstrapping with 1000 replicates. Multiple alignment of nucleotide/amino acid sequences determined in the present study and those retrieved from the GenBank database was performed by the Clustal Omega program https://www.ebi.ac.uk/Tools/msa/clustalo/ (accessed on 20 December 2020), which was also used for calculation of sequence identity. All the sequence data of *S. argenteus* genes (*lukS-PV/lukF-PV*, *sea*, *seb*, *sec*, *egc-2* cluster, *selx*, *sey*, *selz*, *sel26*, *sel27*, *tst-1*, *blaZ*, SCC*mec*, *erm(C)*, *msr(A)*, *lnu(A)*, *tet(K)*, *aac(6′)-Ie-aph(2″)-Ia*, *gyrA/B*, *parC/E*) determined in this study were deposited in the GenBank database under the accession numbers shown in Table S6.

### 4.5. Statistical Analysis

Statistical analyses were performed using IBM SPSS Statistics ver.26. The Chi-square test was used to analyze the differences in prevalence of bacterial attribute information among identified STs. A *p*-value < 0.05 was considered statistically significant.

## Figures and Tables

**Table 1:** Specimens and prevalence of virulence factors in different sequence types (STs) of *S. argenteus* isolates (*n* = 82).

Specimens, Virulence Factor (-Associated) Genes	Number of Isolates (%)			
	ST1223 (*n* = 25)	ST2198 (*n* = 20)	ST2250 (*n* = 37)	Total (*n* = 82)
specimens				
wound	1 (4)	0 (0)	0 (0)	1 (1.2)
pus	0 (0)	1 (5)	1 (2.7)	2 (2.4)
ear pus	3 (12)	2 (10)	2 (5.4)	7 (8.5)
ear discharge	2 (8)	3 (15)	5 (13.5)	10 (12.2)
nasal discharge	0 (0)	4 (20)	7 (18.9)	11 (13.4)
pharynx	4 (16)	0 (0)	2 (5.4)	6 (7.3)
sputum	6 (24)	5 (25)	5 (13.5)	16 (19.5)
stool	3 (12)	2 (10)	7 (18.9)	12 (14.6)
urine	3 (12)	0 (0)	4 (10.8)	7 (8.5)
punctured fluid	1 (4)	1 (5)	0 (0)	2 (2.4)
vaginal discharge	1 (4)	2 (10)	1 (2.7)	4 (4.9)
intestinal juice	1 (4)	0 (0)	0 (0)	1 (1.2)
blood	0 (0)	0 (0)	1 (2.7)	1 (1.2)
skin	0 (0)	0 (0)	1 (2.7)	1 (1.2)
tongue coating	0 (0)	0 (0)	1 (2.7)	1 (1.2)
Leukocidin, haemolysins, enterotoxins, TSST-1 ^1^				
*hla*	25 (100)	20 (100)	37 (100)	82 (100)
*hlb*	25 (100)	20 (100)	37 (100)	82 (100)
*hld*	25 (100)	20 (100)	37 (100)	82 (100)
*hlg*	20 (80)	19 (95)	32 (86.5)	71 (86.6)
*lukS-PV-lukF-PV*	0 (0)	0 (0)	1 (2.7)	1 (1.2)
*sea*	0 (0)	1 (5)	0 (0)	1 (1.2)
*seb*	15 (60) *	0 (0)	0 (0)	15 (18.3)
*sec*	0 (0)	0 (0)	4 (10.8)	4 (4.9)
*egc-2 (seg-sei-sem-sen-seo-seu)*	25 (100) *	0 (0)	0 (0)	25 (30.5)
*selx*	6 (24)	12 (60) *	2 (5.4)	20 (24.4)
*sey*	0 (0)	0 (0)	37 (100) *	37 (45.1)
*selw*	21 (84) *	0 (0)	0 (0)	21 (25.6)
*selz*	8 (32)	0 (0)	11 (29.7)	19 (23.2)
*sel26-sel27*	0 (0)	0 (0)	25 (67.6) *	25 (30.5)
*tst-1*	0 (0)	0 (0)	4 (10.8)	4 (4.9)
Adhesins, modulators of host defense ^1^				
*ebpS*	25 (100)	20 (100)	37 (100)	82 (100)
*fnbA*	25 (100)	20 (100)	37 (100)	82 (100)
*fnbB*	25 (100)	20 (100)	37 (100)	82 (100)
*clfB*	20 (80)	16 (80)	32 (86.5)	68 (82.9)
*eno*	21 (84)	18 (90)	35 (94.6)	74 (90.2)
*cna*	19 (76)	12 (60)	26 (70.3)	57 (69.5)
*icaA*	19 (76)	17 (85)	27 (73)	63 (76.8)
*sdrC*	18 (72) *	3 (15)	21 (56.8)	42 (51.2)
*sdrD*	14 (56)	9 (45)	25 (67.6)	48 (58.5)
*sdrE*	3 (12)	9 (45)	20 (54.1) *	32 (39)
*sak*, *chp*, *scn*	0 (0)	7 (35) *	0 (0)	7 (8.5)
*sak*, *scn* (*chp*-negative)	0 (0)	0 (0)	25 (67.6) *	25 (30.5)
*scn* (*sak*-, *chp*-negative)	7 (28)*	0 (0)	0 (0)	7 (8.5)

^1^ The following genes were not detected in any isolate: *lukM*, *lukDE*, *sed*, *see*, *seh*, *sep*, *seq*, *ser*, *ses*, *set*, *eta*, *etb*, *etd*, *fib*, *clfA*, *icaD*, *bap*, *edn-A*, *edn-B*, *vWbp*. * *p* < 0.01.

**Table 2:** Prevalence of antimicrobial resistance and resistance genes in different STs of *S. argenteus* isolates (*n* = 82).

Antimicrobials, Resistance Genes	Number of Isolates (%)			
	ST1223 (*n* = 25)	ST2198 (*n* = 20)	ST2250 (*n* = 37)	Total (*n* = 82)
Antimicrobials ^1^				
OXA	0 (0)	0 (0)	1 (2.7)	1 (1.2)
FOX	0 (0)	0 (0)	1 (2.7)	1 (1.2)
AMP	1 (4)	12 (60) **	3 (8.1)	16 (19.5)
ERY	0 (0)	7 (35) **	0 (0)	7 (8.5)
CLA	0 (0)	7 (35) **	0 (0)	7 (8.5)
AZM	0 (0)	7 (35) **	0 (0)	7 (8.5)
LIN	0 (0)	5 (25) **	0 (0)	5 (6.1)
CLI	0 (0)	1 (5)	0 (0)	1 (1.2)
TET	0 (0)	0 (0)	1 (2.7)	1 (1.2)
GEN	1 (4)	5 (25) **	0 (0)	6 (7.3)
KAN	1 (4)	5 (25) *	2 (5.4)	8 (9.8)
ABK, CFZ, CMZ, DOX, Q-D, V-G, FMX, FOF, IPM, LVX, LZD, MIN, SXT, TEC, VAN	0 (0)	0 (0)	0 (0)	0 (0)
Drug resistance genes ^2^				
*mecA*	0 (0)	0 (0)	1 (2.7)	1 (1.2)
*blaZ*	1 (4)	12 (60) **	3 (8.1)	16 (19.5)
*erm(C)*	0 (0)	1 (5)	0 (0)	1 (1.2)
*msr(A)*	0 (0)	5 (25) **	0 (0)	5 (6.1)
*lnu(A)*	0 (0)	4 (20) **	0 (0)	4 (4.9)
*tet(K)*	0 (0)	0 (0)	1 (2.7)	1 (1.2)
*aac(6′)-Ie-aph(2″)-Ia*	1 (4)	5 (25) **	0 (0)	6 (7.3)
*aph(3′)-IIIa*	0 (0)	0 (0)	2 (5.4)	2 (2.4)

^1^ Antimicrobials tested: ABK, Arbekacin; AMP, ampicillin; AZM, Azithromycin; CFZ, Cefazolin; CLA, Clarithromycin; CLI, Clindamycin; CMZ, Cefmetazole; DOX, doxycycline; ERY, Erythormycin; FMX, Flomoxef: FOF, Fosfomycin; FOX, Cefoxitin; GEN, Gentamicin; IPM, Imipenem; KAN, Kanamycin; LIN, Lincomycin; LVX, Levofloxacin; LZD, Linezolid; MIN, Minocycline; OXA, Oxacillin; Q-D, Quinopristin-Dalfopristin; SXT, Sulfamethoxazole-Trimethoprim; TEC, Teicoplanin; TET, tetracycline; VAN, Vancomycin;V-G, Virginiamycin/Pristinamycin. ^2^ The following genes were not detected in any isolate: *erm(A)*, *erm*(*B*), *erm(F)*, *erm(T)*, *erm(X)*, *erm(Y)*, *tet(M)*, *tet(L)*, *lnu(B)*, *optrA*, *cfr*, *aac(6′)-Im*, *ant(4′)-Ia*, *ant(6)-Ia*, *ant(9)-Ia*, *ant(9)-Ib*, *ant(3″)-Ia*, *aph(2″)-Ib*, *aph(2″)-Ic*, *aph(2″)-Id*, *aph(2″)-Ie*, *lsa(A)*, *lsa(E)*, *vatA*, *vatB*, *vatC*, *vatD*, *vatE*, *vatG*, *vgaA*, *vgaB*, *vgaD*, *vgbA*, *vgb* and, *mefAE*. * *p* < 0.05; ** *p* < 0.01.

## Data Availability

Not applicable.

## Supplementary Materials

The following are available online at https://www.mdpi.com/2076-0817/10/2/163/s1, Figure S1: Alignment of nucleotide (a) and amino acid (b) sequences of SEB, including nucleotide variants v1-v6 and amino acid variants v1-v4, Figure S2: Alignment of *repL*, *erm(C)* and its promoter region of *S. aureus* strain USA300_FPR3757 (plasmid pSA03) and USA300-SUR11 (plasmid pUSA05-1-SUR11), *S. argenteus* strain SaM260_MSHR and SG70 (present study), Figure S3: Alignment of partial GyrA, GyrB, ParC, and ParE amino acid sequences including quinolone-resistance determining regions (QRDR) of *S. argenteus* strains (SG95, SG99, SG103, XNO106, and MSHR1132) and *S. aureus* strains (N315 and NCTC8325) that are fluoroquinolone-susceptible. Table S1: Monthly incidence of three STs of *S. argenteus*, Table S2: Drug resistance profile and prevalence of virulence factors/drug resistance genes in 82 *S. argenteus* isolates in Hokkaido, Japan for one year-period (August 2019–July 2020), Table S3: Sequence identity of component genes (*seg-sei-sem-sen-seo-seu*) in *egc-2* of *S. argenteus* SG48 in the present study to those of *S. argenteus*, *S. aureus* and *S. schweitzeri* isolates reported previously, Table S4: Nucleotide sequence identity of drug resistance genes detected in the present study to those of *S.aureus* and other staphylococcal species and enterococcus retrieved from GenBank database, Table S5: Primers used in this study, Table S6: GenBank accession numbers assigned to *lukS-PV-lukF-PV*, *sea*, *seb*, *sec*, *egc-2*, *tst-1*, *blaZ*, SCC*mec* complex, *erm(C)*, *msr(A)*, *lnu(A)*, *tet(K)*, *aac(6′)-Ie-aph(2″)-Ia*, *gyrA/B*, *parC/E* genes detected in the present study.
