# Supplementary material for: Distribution of Virulence Factors and Resistance Determinants in Three Genotypes of Staphylococcus argenteus Clinical Isolates in Japan
Source: Pathogens. 2021 Feb 3;10(2):163. doi: 10.3390/pathogens10020163 (PMC7913748; doi:10.3390/pathogens10020163)
Supplement: Supplementary file 1 [file pathogens-10-00163-s001.zip › Suppl-20210129/Figure-S1.docx]

**(a)**

KLT6-seb-v1(KX168628) ATGTATAAGAGATTATTTATTTCACATGTAATTTTGATATTCGCACTGATATTAGTTATT 60

SANC14-seb-v2(KX168629) ATGTATAAGAGATTATTTATTTCACATGTAATTTTGATATTCGTACTGATATTAGTTATT 60

SAI10-seb-v3(KX168630) ATGTATAATAGATTATTTGTTTCACGTGTAATTTTGATATTCGCACTGATACTAGTTATT 60

SG39 (seb-v3) ATGTATAATAGATTATTTGTTTCACGTGTAATTTTGATATTCGCACTGATACTAGTTATT 60

SAI45-seb-v4(KX168631) ATGTATAAGAGATTATTTATTTCACATGTAATTTTGATATTCGTACTGATATTAGTTATT 60

RKI4-seb-v5(KX168632) ATGTATAATAGATTATTTGTTTCACGTGTAATTTTGATATTCGCACTGATACTAGTTATT 60

SG63 (seb-v6) ATGTATAATAGATTATTTGTTTCACGTGTAATTTTGATATTCGCACTGATACTAGTTATT 60

******** ********* ****** ***************** ******* ********

KLT6-seb-v1(KX168628) TCTACACCCAACGTTTTAGCAGAGAGTCAACCAGATCCTAAACCAGATGAGTTGCACAAA 120

SANC14-seb-v2(KX168629) TCTACACCCAACGTTTTAGCAGAGAGTCAACCAGATCCTAAACCAGATGAGTTGCACAAA 120

SAI10-seb-v3(KX168630) TATACACCCAACGTTTTAGCAGAAAGCCAACCAGATCCTAAACCAGATGAGTTGCACAAA 120

SG39 TATACACCCAACGTTTTAGCAGAAAGCCAACCAGATCCTAAACCAGATGAGTTGCACAAA 120

SAI45-seb-v4(KX168631) TCTACACCCAACGTTTTAGCAGAGAGTCAACCAGATCCTAAACCAGATGAGTTGCACAAA 120

RKI4-seb-v5(KX168632) TATACACCCAACGTTTTAGCAGAAAGCCAACCAGATCCTAAACCAGATGAGTTGCACAAA 120

SG63 TATACACCCAACGTTTTAGCAGAAAGCCAACCAGATCCTAAACCAGATGAGTTGCACAAA 120

* ********************* ** *********************************

KLT6-seb-v1(KX168628) TCGAGTAAATTCACTGGTTTGATGGAAAATATGAAAGTTTTGTATGATGATAATCATGTA 180

SANC14-seb-v2(KX168629) GCGAGTAAATTCACTGGTTTGATGGAAAATATGAAAGTTTTGTATGATGATAATCATGTA 180

SAI10-seb-v3(KX168630) GCGAGTAAATTCACTGGTTTGATGGAAAATATGAAAGTTTTATACGATGATAATCATGTA 180

SG39 GCGAGTAAATTCACTGGTTTGATGGAAAATATGAAAGTTTTATACGATGATAATCATGTA 180

SAI45-seb-v4(KX168631) GCGAGTAAATTCACTGGTTTGATGGAAAATATGAAAGTTTTGTATGATGATAATCATGTA 180

RKI4-seb-v5(KX168632) GCGAGTAAATTCACTGGTTTGATGGAAAATATGAAAGTTTTATACGATGATAATCATGTA 180

SG63 GCGAGTAAATTCACTGGTTTGATGGAAAATATCAAAGTTTTATACGATGATAATCATGTA 180

******************************* ******** ** ***************

KLT6-seb-v1(KX168628) TCAGCAATAAACGTTAAATCTATAGATCAATTTCTATACTTTGACTTAATATATTCTATT 240

SANC14-seb-v2(KX168629) TCAGCAATAAACGTTAAATCTATAGATCAATTTCTATACTTTGACTTAATATATTCTATT 240

SAI10-seb-v3(KX168630) TCAGCAATAAACGTTAAATCTATAGATCAATTTCTATACTTTGACTTAATATATTCTATT 240

SG39 TCAGCAATAAACGTTAAATCTATAGATCAATTTCTATACTTTGACTTAATATATTCTATT 240

SAI45-seb-v4(KX168631) TCAGCAATAAACGTTAAATCTATAGATCAATTTCTATACTTTGACTTAATATATTCTATT 240

RKI4-seb-v5(KX168632) TCAGCAATAAACGTTAAATCTATAGATCAATTTCTATACTTTGACTTAATATATTCTATT 240

SG63 TCAGCAATAAACGTTAAATATATAGATCAATTTCTATACTTTGACTTAATATATTCTATT 240

******************* ****************************************

KLT6-seb-v1(KX168628) AAGGACACTAAGTTAGGGAATTATGATAATGTTCGAGTCGAATTTAAAAACAAAGATTTA 300

SANC14-seb-v2(KX168629) AAGGACACTAAGTTAGGGAATTATGATAATGTTCGAGTCGAATTTAAAAACAAAGATTTA 300

SAI10-seb-v3(KX168630) AAGGACACTAAGTTAGGGAATTATGATAATGTTCGAGTCGAATTTAAAAACAAAGATTTA 300

SG39 AAGGACACTAAGTTAGGGAATTATGATAATGTTCGAGTCGAATTTAAAAACAAAGATTTA 300

SAI45-seb-v4(KX168631) AAGGACACTAAGTTAGGGAATTATGATAATGTTCGAGTCGAATTTAAAAACAAAGATTTA 300

RKI4-seb-v5(KX168632) AAGGACACTAAGTTAGGGAATTATGATAATGTTCGAGTCGAATTTAAAAACAAAGATTTA 300

SG63 AAGGACACTAAGTTAGGGAATTATGATAATGTTCGAGTCGAATTTAAAAACAAAGATTTA 300

************************************************************

KLT6-seb-v1(KX168628) GCTGATAAATACAAAGATAAATACGTAGATGTGTTTGGAGCTAATTATTATTATCAATGT 360

SANC14-seb-v2(KX168629) GCTGATAAATACAAAGATAAATACGTAGATGTGTTTGGAGCTAATTATTACTATCAATGT 360

SAI10-seb-v3(KX168630) GCTGATAAATACAAAGATAAATACGTAGATGTGTTTGGAGCTAATTATTATTATCAATGT 360

SG39 GCTGATAAATACAAAGATAAATACGTAGATGTGTTTGGAGCTAATTATTATTATCAATGT 360

SAI45-seb-v4(KX168631) GCTGATAAATACAAAGATAAATACGTAGATGTGTTTGGAGCTAATTATTACTATCAATGT 360

RKI4-seb-v5(KX168632) GCTGATAAATACAAAGATAAATACGTAGATGTGTTTGGAGCTAATTATTATTATCAATGT 360

SG63 GCTGATAAATACAAAGATAAATACGTAGATGTGTTTGGAGCTAATTATTATTATCAATGT 360

************************************************** *********

KLT6-seb-v1(KX168628) TATTTTTCTAAAAAAACGAATGATATTAATTCGCATCAAACTGACAAACGAAAAACTTGT 420

SANC14-seb-v2(KX168629) TATTTTTCTAAAAAAACGAATGATATTAATTCACATCAAACTGACAAACGAAAAACTTGT 420

SAI10-seb-v3(KX168630) TATTTTTCTAAAAAAACGAATGATATTAATTCACATCAAACTGATAAACGAAAAACTTGT 420

SG39 TATTTTTCTAAAAAAACGAATGATATTAATTCACATCAAACTGATAAACGAAAAACTTGT 420

SAI45-seb-v4(KX168631) TATTTTTCTAAAAAAACGAATGATATTAATTCACATCAAACTGACAAACGAAAAACTTGT 420

RKI4-seb-v5(KX168632) TATTTTTCTAAAAAAACGAATGATATTAATTCACATCAAACTGACAAACGAAAAACTTGT 420

SG63 TATTTTTCTAAAAAAACGAATGATATTAATTCACATCAAACTGATAAACGAAAAACTTGT 420

******************************** *********** ***************

KLT6-seb-v1(KX168628) ATGTATGGTGGTGTAACTGAGCATAATGGAAACCAATTAGATAAATATAGAAGTATTACT 480

SANC14-seb-v2(KX168629) ATGTATGGTGGTGTAACTGAGCATAATGGAAACCAATTAGATAAATATAGAAGTATTACT 480

SAI10-seb-v3(KX168630) ATGTATGGTGGTGTAACTGAGCATAATGGAAACCATTTAGATAAATATAGAAGTATTACT 480

SG39 ATGTATGGTGGTGTAACTGAGCATAATGGAAACCATTTAGATAAATATAGAAGTATTACT 480

SAI45-seb-v4(KX168631) ATGTATGGTGGTGTAACTGAGCATAATGGAAACCAATTAGATAAATATAGAAGTATTACT 480

RKI4-seb-v5(KX168632) ATGTATGGTGGTGTAACTGAGCATAATGGAAACCATTTAGATAAATATAGAAGTATTACT 480

SG63 ATGTATGGTGGTGTAACTGAGCATAATGGAAACCATTTAGATAAATATAGAAGTATTACT 480

*********************************** ************************

KLT6-seb-v1(KX168628) GTTCGGGTATTTGAAGATGGTAAAAATTTATTATCTTTTGACGTACAAACTAATAAGAAA 540

SANC14-seb-v2(KX168629) GTTCGGGTATTTGAAGATGGTAAAAATTTATTATCTTTTGACGTACAAACTAATAAGAAA 540

SAI10-seb-v3(KX168630) GTTAGGGTATTTGAAGATGGTAAAAATTTATTGTCTTTTGACGTACAAACTAATAAGAAA 540

SG39 GTTAGGGTATTTGAAGATGGTAAAAATTTATTGTCTTTTGACGTACAAACTAATAAGAAA 540

SAI45-seb-v4(KX168631) GTTAGGGTATTTGAAGATGGTAAAAATTTATTATCTTTTGACGTACAAACTAATAAGAAA 540

RKI4-seb-v5(KX168632) GTTAGGGTATTTGAAGATGGTAAAAATTTATTGTCTTTTGACGTACAAACTAATAAGAAA 540

SG63 GTTAGGGTATTTGAAGATGGTAAAAATTTATTGTCTTTTGACGTACAAACTAATAAGAAA 540

*** **************************** ***************************

KLT6-seb-v1(KX168628) AAGGTGACTGCTCAAGAATTAGATTACCTAACTCGTCACTATTTGGTGAAAAATAAAAAA 600

SANC14-seb-v2(KX168629) AAAGTGACTGCTCAAGAATTAGATTACCTAACTCGTCACTATTTGGTGAAAAATAAAAAA 600

SAI10-seb-v3(KX168630) AAAGTGACTGCTCAAGAATTAGATTACCTAACTCGTCACTATTTGGTGAAAAATAAAAAA 600

SG39 AAAGTGACTGCTCAAGAATTAGATTACCTAACTCGTCACTATTTGGTGAAAAATAAAAAA 600

SAI45-seb-v4(KX168631) AAAGTGACTGCTCAAGAATTAGATTACCTAACTCGTCACTATTTGGTGAAAAATAAAAAA 600

RKI4-seb-v5(KX168632) AAAGTGACTGCTCAAGAATTAGATTACCTAACTCGTCACTATTTGGTGAAAAATAAAAAA 600

SG63 AAAGTGACTGCTCAAGAATTAGATTACCTAACTCGTCACTATTTGGTGAAAAATAAAAAA 600

** *********************************************************

KLT6-seb-v1(KX168628) CTCTATGAATTTAACAACTCGCCTTATGAAACGGGATATATTAAATTTATAGAAAATGAG 660

SANC14-seb-v2(KX168629) CTCTATGAATTTAACAACTCGCCTTATGAAACGGGATATATTAAATTTATAGAAAGTGAG 660

SAI10-seb-v3(KX168630) CTCTATGAATTTAACAACTCACCTTATGAAACGGGATATATTAAATTTATAGAAAGTGAG 660

SG39 CTCTATGAATTTAACAACTCACCTTATGAAACGGGATATATTAAATTTATAGAAAGTGAG 660

SAI45-seb-v4(KX168631) CTCTATGAATTTAACAACTCGCCTTATGAAACGGGATATATTAAATTTATAGAAAGTGAG 660

RKI4-seb-v5(KX168632) CTCTATGAATTTAACAACTCACCTTATGAAACGGGATATATTAAATTTATAGAAAGTGAG 660

SG63 CTCTATGAATTTAACAACTCACCTTATGAAACGGGATATATTAAATTTATAGAAAGTGAG 660

******************** ********************************** ****

KLT6-seb-v1(KX168628) AATAGCTTTTGGTATGACATGATGCCTGCACCAGGAGATAAATTTGACCAATCTAAATAT 720

SANC14-seb-v2(KX168629) AATAGCTTTTGGTATGACATGATGCCTGCACCAGGAGATAAATTTGACCAATCTAAATAT 720

SAI10-seb-v3(KX168630) AATAGCTTTTGGTATGACATGATGCCTGCACCAGGAGATAAATTTGACCAATCTAAATAT 720

SG39 AATAGCTTTTGGTATGACATGATGCCTGCACCAGGAGATAAATTTGACCAATCTAAATAT 720

SAI45-seb-v4(KX168631) AATAGCTTTTGGTATGACATGATGCCTGCACCAGGAGATAAATTTGACCAATCTAAATAT 720

RKI4-seb-v5(KX168632) AATAGCTTTTGGTATGACATGATGCCTGCACCAGGAGATAAATTTGACCAATCTAAATAT 720

SG63 AATAGCTTTTGGTATGACATGATGCCTGCACCAGGAGATAAATTTGACCAATCTAAATAT 720

************************************************************

KLT6-seb-v1(KX168628) TTAATGATGTACAATGACAATAAAATGGTTGATTCTAAAGATGTGAAGATTGAAGTTTAT 780

SANC14-seb-v2(KX168629) TTAATGATGTACAATGATAATAAATTGGTTGATTCTAAAGATGTGAAGATTGAAGTTTAT 780

SAI10-seb-v3(KX168630) TTAATGATGTACAATGATAATAAATTGGTTGATTCTAAAGATGTGAAGATTGAAGTTTAT 780

SG39 TTAATGATGTACAATGATAATAAATTGGTTGATTCTAAAGATGTGAAGATTGAAGTTTAT 780

SAI45-seb-v4(KX168631) TTAATGATGTACAATGATAATAAATTGGTTGATTCTAAAGATGTGAAGATTGAAGTTTAT 780

RKI4-seb-v5(KX168632) TTAATGATGTACAATGATAATAAATTGGTTGATTCTAAAGATGTGAAGATTGAAGTTTAT 780

SG63 TTAATGATGTACAATGATAATAAATTGGTTGATTCTAAAGATGTGAAGATTGAAGTTTAT 780

***************** ****** ***********************************

KLT6-seb-v1(KX168628) CTTACGACAAAGAAAAAGTGA 801

SANC14-seb-v2(KX168629) CTTACGACAAAGAAAAAGTGA 801

SAI10-seb-v3(KX168630) CTTACGACAAAGAAAAAGTGA 801

SG39 CTTACGACAAAGAAAAAGTGA 801

SAI45-seb-v4(KX168631) CTTACGACAAAGAAAAAGTGA 801

RKI4-seb-v5(KX168632) CTTACGACAAAGAAAAAGTGA 801

SG63 CTTACGACAAAGAAAAAGTGA 801

*********************

**(b)**

KLT6-seb-aa-v1(KX168628) MYKRLFISHVILIFALILVISTPNVLAESQPDPKPDELHKSSKFTGLMENMKVLYDDNHV 60

SAI45-seb-aa-v2(KX168631) MYKRLFISHVILIFVLILVISTPNVLAESQPDPKPDELHKASKFTGLMENMKVLYDDNHV 60

RKI4-seb-aa-v3(KX168632) MYNRLFVSRVILIFALILVIYTPNVLAESQPDPKPDELHKASKFTGLMENMKVLYDDNHV 60

SA15-03-S.argenteus(LC275973) MYNRLFVSRVILIFALILVIYTPNVLAESQPDPKPDELHKASKFTGLMENMKVLYDDNHV 60

SG39 (seb-aa-v3) MYNRLFVSRVILIFALILVIYTPNVLAESQPDPKPDELHKASKFTGLMENMKVLYDDNHV 60

SG40 (seb-aa-v3) MYNRLFVSRVILIFALILVIYTPNVLAESQPDPKPDELHKASKFTGLMENMKVLYDDNHV 60

SG42 (seb-aa-v3) MYNRLFVSRVILIFALILVIYTPNVLAESQPDPKPDELHKASKFTGLMENMKVLYDDNHV 60

SG48 (seb-aa-v3) MYNRLFVSRVILIFALILVIYTPNVLAESQPDPKPDELHKASKFTGLMENMKVLYDDNHV 60

SG57 (seb-aa-v3) MYNRLFVSRVILIFALILVIYTPNVLAESQPDPKPDELHKASKFTGLMENMKVLYDDNHV 60

SG73 (seb-aa-v3) MYNRLFVSRVILIFALILVIYTPNVLAESQPDPKPDELHKASKFTGLMENMKVLYDDNHV 60

SG83 (seb-aa-v3) MYNRLFVSRVILIFALILVIYTPNVLAESQPDPKPDELHKASKFTGLMENMKVLYDDNHV 60

SG87 (seb-aa-v3) MYNRLFVSRVILIFALILVIYTPNVLAESQPDPKPDELHKASKFTGLMENMKVLYDDNHV 60

SG95 (seb-aa-v3) MYNRLFVSRVILIFALILVIYTPNVLAESQPDPKPDELHKASKFTGLMENMKVLYDDNHV 60

SG103 (seb-aa-v3) MYNRLFVSRVILIFALILVIYTPNVLAESQPDPKPDELHKASKFTGLMENMKVLYDDNHV 60

SG63 (seb-aa-v4) MYNRLFVSRVILIFALILVIYTPNVLAESQPDPKPDELHKASKFTGLMENIKVLYDDNHV 60

**:***:*:*****.***** *******************:*********:*********

KLT6-seb-aa-v1(KX168628) SAINVKSIDQFLYFDLIYSIKDTKLGNYDNVRVEFKNKDLADKYKDKYVDVFGANYYYQC 120

SAI45-seb-aa-v2(KX168631) SAINVKSIDQFLYFDLIYSIKDTKLGNYDNVRVEFKNKDLADKYKDKYVDVFGANYYYQC 120

RKI4-seb-aa-v3(KX168632) SAINVKSIDQFLYFDLIYSIKDTKLGNYDNVRVEFKNKDLADKYKDKYVDVFGANYYYQC 120

SA15-03-S.argenteus(LC275973) SAINVKSIDQFLYFDLIYSIKDTKLGNYDNVRVEFKNKDLADKYKDKYVDVFGANYYYQC 120

SG39 SAINVKSIDQFLYFDLIYSIKDTKLGNYDNVRVEFKNKDLADKYKDKYVDVFGANYYYQC 120

SG40 SAINVKSIDQFLYFDLIYSIKDTKLGNYDNVRVEFKNKDLADKYKDKYVDVFGANYYYQC 120

SG42 SAINVKSIDQFLYFDLIYSIKDTKLGNYDNVRVEFKNKDLADKYKDKYVDVFGANYYYQC 120

SG48 SAINVKSIDQFLYFDLIYSIKDTKLGNYDNVRVEFKNKDLADKYKDKYVDVFGANYYYQC 120

SG57 SAINVKSIDQFLYFDLIYSIKDTKLGNYDNVRVEFKNKDLADKYKDKYVDVFGANYYYQC 120

SG73 SAINVKSIDQFLYFDLIYSIKDTKLGNYDNVRVEFKNKDLADKYKDKYVDVFGANYYYQC 120

SG83 SAINVKSIDQFLYFDLIYSIKDTKLGNYDNVRVEFKNKDLADKYKDKYVDVFGANYYYQC 120

SG87 SAINVKSIDQFLYFDLIYSIKDTKLGNYDNVRVEFKNKDLADKYKDKYVDVFGANYYYQC 120

SG95 SAINVKSIDQFLYFDLIYSIKDTKLGNYDNVRVEFKNKDLADKYKDKYVDVFGANYYYQC 120

SG103 SAINVKSIDQFLYFDLIYSIKDTKLGNYDNVRVEFKNKDLADKYKDKYVDVFGANYYYQC 120

SG63 SAINVKYIDQFLYFDLIYSIKDTKLGNYDNVRVEFKNKDLADKYKDKYVDVFGANYYYQC 120

****** *****************************************************

KLT6-seb-aa-v1(KX168628) YFSKKTNDINSHQTDKRKTCMYGGVTEHNGNQLDKYRSITVRVFEDGKNLLSFDVQTNKK 180

SAI45-seb-aa-v2(KX168631) YFSKKTNDINSHQTDKRKTCMYGGVTEHNGNQLDKYRSITVRVFEDGKNLLSFDVQTNKK 180

RKI4-seb-aa-v3(KX168632) YFSKKTNDINSHQTDKRKTCMYGGVTEHNGNHLDKYRSITVRVFEDGKNLLSFDVQTNKK 180

SA15-03-S.argenteus(LC275973) YFSKKTNDINSHQTDKRKTCMYGGVTEHNGNHLDKYRSITVRVFEDGKNLLSFDVQTNKK 180

SG39 YFSKKTNDINSHQTDKRKTCMYGGVTEHNGNHLDKYRSITVRVFEDGKNLLSFDVQTNKK 180

SG40 YFSKKTNDINSHQTDKRKTCMYGGVTEHNGNHLDKYRSITVRVFEDGKNLLSFDVQTNKK 180

SG42 YFSKKTNDINSHQTDKRKTCMYGGVTEHNGNHLDKYRSITVRVFEDGKNLLSFDVQTNKK 180

SG48 YFSKKTNDINSHQTDKRKTCMYGGVTEHNGNHLDKYRSITVRVFEDGKNLLSFDVQTNKK 180

SG57 YFSKKTNDINSHQTDKRKTCMYGGVTEHNGNHLDKYRSITVRVFEDGKNLLSFDVQTNKK 180

SG73 YFSKKTNDINSHQTDKRKTCMYGGVTEHNGNHLDKYRSITVRVFEDGKNLLSFDVQTNKK 180

SG83 YFSKKTNDINSHQTDKRKTCMYGGVTEHNGNHLDKYRSITVRVFEDGKNLLSFDVQTNKK 180

SG87 YFSKKTNDINSHQTDKRKTCMYGGVTEHNGNHLDKYRSITVRVFEDGKNLLSFDVQTNKK 180

SG95 YFSKKTNDINSHQTDKRKTCMYGGVTEHNGNHLDKYRSITVRVFEDGKNLLSFDVQTNKK 180

SG103 YFSKKTNDINSHQTDKRKTCMYGGVTEHNGNHLDKYRSITVRVFEDGKNLLSFDVQTNKK 180

SG63 YFSKKTNDINSHQTDKRKTCMYGGVTEHNGNHLDKYRSITVRVFEDGKNLLSFDVQTNKK 180

*******************************:****************************

KLT6-seb-aa-v1(KX168628) KVTAQELDYLTRHYLVKNKKLYEFNNSPYETGYIKFIENENSFWYDMMPAPGDKFDQSKY 240

SAI45-seb-aa-v2(KX168631) KVTAQELDYLTRHYLVKNKKLYEFNNSPYETGYIKFIESENSFWYDMMPAPGDKFDQSKY 240

RKI4-seb-aa-v3(KX168632) KVTAQELDYLTRHYLVKNKKLYEFNNSPYETGYIKFIESENSFWYDMMPAPGDKFDQSKY 240

SA15-03-S.argenteus(LC275973) KVTAQELDYLTRHYLVKNKKLYEFNNSPYETGYIKFIESENSFWYDMMPAPGDKFDQSKY 240

SG39 KVTAQELDYLTRHYLVKNKKLYEFNNSPYETGYIKFIESENSFWYDMMPAPGDKFDQSKY 240

SG40 KVTAQELDYLTRHYLVKNKKLYEFNNSPYETGYIKFIESENSFWYDMMPAPGDKFDQSKY 240

SG42 KVTAQELDYLTRHYLVKNKKLYEFNNSPYETGYIKFIESENSFWYDMMPAPGDKFDQSKY 240

SG48 KVTAQELDYLTRHYLVKNKKLYEFNNSPYETGYIKFIESENSFWYDMMPAPGDKFDQSKY 240

SG57 KVTAQELDYLTRHYLVKNKKLYEFNNSPYETGYIKFIESENSFWYDMMPAPGDKFDQSKY 240

SG73 KVTAQELDYLTRHYLVKNKKLYEFNNSPYETGYIKFIESENSFWYDMMPAPGDKFDQSKY 240

SG83 KVTAQELDYLTRHYLVKNKKLYEFNNSPYETGYIKFIESENSFWYDMMPAPGDKFDQSKY 240

SG87 KVTAQELDYLTRHYLVKNKKLYEFNNSPYETGYIKFIESENSFWYDMMPAPGDKFDQSKY 240

SG95 KVTAQELDYLTRHYLVKNKKLYEFNNSPYETGYIKFIESENSFWYDMMPAPGDKFDQSKY 240

SG103 KVTAQELDYLTRHYLVKNKKLYEFNNSPYETGYIKFIESENSFWYDMMPAPGDKFDQSKY 240

SG63 KVTAQELDYLTRHYLVKNKKLYEFNNSPYETGYIKFIESENSFWYDMMPAPGDKFDQSKY 240

**************************************.*********************

KLT6-seb-aa-v1(KX168628) LMMYNDNKMVDSKDVKIEVYLTTKKK 266

SAI45-seb-aa-v2(KX168631) LMMYNDNKLVDSKDVKIEVYLTTKKK 266

RKI4-seb-aa-v3(KX168632) LMMYNDNKLVDSKDVKIEVYLTTKKK 266

SA15-03-S.argenteus(LC275973) LMMYNDNKLVDSKDVKIEVYLTTKKK 266

SG39 LMMYNDNKLVDSKDVKIEVYLTTKKK 266

SG40 LMMYNDNKLVDSKDVKIEVYLTTKKK 266

SG42 LMMYNDNKLVDSKDVKIEVYLTTKKK 266

SG48 LMMYNDNKLVDSKDVKIEVYLTTKKK 266

SG57 LMMYNDNKLVDSKDVKIEVYLTTKKK 266

SG73 LMMYNDNKLVDSKDVKIEVYLTTKKK 266

SG83 LMMYNDNKLVDSKDVKIEVYLTTKKK 266

SG87 LMMYNDNKLVDSKDVKIEVYLTTKKK 266

SG95 LMMYNDNKLVDSKDVKIEVYLTTKKK 266

SG103 LMMYNDNKLVDSKDVKIEVYLTTKKK 266

SG63 LMMYNDNKLVDSKDVKIEVYLTTKKK 266

********:*****************

**Figure S1.** Alignment of nucleotide (a) and amino acid (b) sequences of SEB, including nucleotide variants v1-v6 and amino acid variants v1-v4. Nucleotide or amino acid which is different from that of v1 is shown in yellow. Asterisk indicates identical nucleotide/amino acid. SEB nucleotide sequence of SG39 was assigned to v3, as well as those of all other *S. argenteus* isolates analyzed (not shown), except for SG63 that was assigned to new variant v6. SEB amino acid sequence of SG63 was classified into new variant v4, while those of *S. argenteus* strain SA15-03 and all other isolates in the present study were assigned to v3.
