# Supplementary material for: Distribution of Virulence Factors and Resistance Determinants in Three Genotypes of Staphylococcus argenteus Clinical Isolates in Japan
Source: Pathogens. 2021 Feb 3;10(2):163. doi: 10.3390/pathogens10020163 (PMC7913748; doi:10.3390/pathogens10020163)
Supplement: Supplementary file 1 [file pathogens-10-00163-s001.zip › Suppl-20210129/Figure-S2.docx]

|→ ***repL***

M260_MSHR ATGAAAGAAAGATATGGAACAGTCTATAAAGGCTCTCAGAGGCTCATAGACGAAGAAAGT 60

pUSA05-1-SUR11 ATGAAAGAAAGATATGGAACAGTCTATAAAGGCTCTCAGAGGCTCATAGACGAAGAAAGT 60

pUSA03 ATGAAAGAAAGATATGGAACAGTCTATAAAGGCTCTCAGAGGCTCATAGACGAAGAAAGT 60

SG70 ATGAAAGAAAGATATGGAACAGTCTATAAAGGCTCTCAGAGGCTCATAGACGAAGAAAGT 60

************************************************************

M260_MSHR GGAGAAGTCATAGAGGTAGACAAGTTATACCGTAAACAAACGTCTGGTAACTTCGTAAAG 120

pUSA05-1-SUR11 GGAGAAGTCATAGAGGTAGACAAGTTATACCGTAAACAAACGTCTGGTAACTTCGTAAAG 120

pUSA03 GGAGAAGTCATAGAGGTAGACAAGTTATACCGTAAACAAACGTCTGGTAACTTCGTAAAG 120

SG70 GGAGAAGTCATAGAGGTAGACAAGTTATACCGTAAACAAACGTCTGGTAACTTCGTAAAG 120

************************************************************

M260_MSHR GCATATATAGTGCAATTAATAAGTATGTTAGATATGATTGGCGGAAAAAAACTTAAAATC 180

pUSA05-1-SUR11 GCATATATAGTGCAATTAATAAGTATGTTAGATATGATTGGCGGAAAAAAACTTAAAATC 180

pUSA03 GCATATATAGTGCAATTAATAAGTATGTTAGATATGATTGGCGGAAAAAAACTTAAAATC 180

SG70 GCATATATAGTGCAATTAATAAGTATGTTAGATATGATTGGCGGAAAAAAACTTAAAATC 180

************************************************************

M260_MSHR GTTAACTATATCCTAGATAATGTCCACTTAAGTAACAATACAATGATAGCTACAACAAGA 240

pUSA05-1-SUR11 GTTAACTATATCCTAGATAATGTCCACTTAAGTAACAATACAATGATAGCTACAACAAGA 240

pUSA03 GTTAACTATATCCTAGATAATGTCCACTTAAGTAACAATACAATGATAGCTACAACAAGA 240

SG70 GTTAACTATATCCTAGATAATGTCCACTTAAGTAACAATACAATGATAGCTACAACAAGA 240

************************************************************

M260_MSHR GAAATAGCAAAAGCTACAGGAACAAGTCTACAAACAGTAATAACAACACTTAAAATCTTA 300

pUSA05-1-SUR11 GAAATAGCAAAAGCTACAGGAACAAGTCTACAAACAGTAATAACAACACTTAAAATCTTA 300

pUSA03 GAAATAGCAAAAGCTACAGGAACAAGTCTACAAACAGTAATAACAACACTTAAAATCTTA 300

SG70 GAAATAGCAAAAGCTACAGGAACAAGTCTACAAACAGTAATAACAACACTTAAAATCTTA 300

************************************************************

M260_MSHR GAAGAAGGAAATATTATAAAAAGAAAAACTGGAGTATTAATGTTAAACCCTGAACTACTA 360

pUSA05-1-SUR11 GAAGAAGGAAATATTATAAAAAGAAAAACTGGAGTATTAATGTTAAACCCTGAACTACTA 360

pUSA03 GAAGAAGGAAATATTATAAAAAGAAAAACTGGAGTATTAATGTTAAACCCTGAACTACTA 360

SG70 GAAGAAGGAAATATTATAAAAAGAAAAACTGGAGTATTAATGTTAAACCCTGAACTACTA 360

************************************************************

M260_MSHR ATGAGAGGCGACGACCAAAAACAAAAATACCTCTTACTCGAATTTGGGAACTTTGAGCAA 420

pUSA05-1-SUR11 ATGAGAGGCGACGACCAAAAACAAAAATACCTCTTACTCGAATTTGGGAACTTTGAGCAA 420

pUSA03 ATGAGAGGCGACGACCAAAAACAAAAATACCTCTTACTCGAATTTGGGAACTTTGAGCAA 420

SG70 ATGAGAGGCGACGACCAAAAACAAAAATACCTCTTACTCGAATTTGGGAACTTTGAGCAA 420

************************************************************

M260_MSHR GAGGCAAATGAAAAACAAGAAAATGCATTATCTGATTATTATTCTTTCAAGGACTAGTAT 480

pUSA05-1-SUR11 GAGGCAAATGAAAAACAAGAAAATGCATTATCTGATTATTATTCTTTCAAGGACTAGTAT 480

pUSA03 GAGGCAAATGAAAAACAAGAAAATGCATTATCTGATTATTATTCTTTCAAGGACTAGTAT 480

SG70 GAGGCAAATGAAAAACAAGAAAATGCATTATCTGATTATTATTCTTTCAAGGACTAGTAT 480

************************************************************

M260_MSHR AACATAAAATCGTCTACAAATAGACAAAAAACCTGCACGCTTAATGTAGATCAAAAGCTT 540

pUSA05-1-SUR11 AACATAAAATCGTCTACAAATAGACAAAAAACCTGCACGCTTAATGTAGATCAAAAGCTT 540

pUSA03 AACATAAAATCGTCTACAAATAGACAAAAAACCTGCACGCTTAATGTAGATCAAAAGCTT 540

SG70 AACATAAAATCGTCTACAAATAGACAAAAAACCTGCACGCTTAATGTAGATCAAAAGCTT 540

************************************************************

M260_MSHR AACGCAAATGAAATAGATTGACCTCCCAATAACACCACGTAGTTATTGGGAGGTCAATCT 600

pUSA05-1-SUR11 AACGCAAATGAAATAGATTGACCTCCCAATAACACCACGTAGTTATTGGGAGGTCAATCT 600

pUSA03 AACGCAAATGAAATAGATTGACCTCCCAATAACACCACGTAGTTATTGGGAGGTCAATCT 600

SG70 AACGCAAATGAAATAGATTGACCTCCCAATAACACCACGTAGTTATTGGGAGGTCAATCT 600

************************************************************

M260_MSHR ATGAAATGCGATTAAGCTTTTTCTAATTCACATAAGCGTGCAGGTTTAAAGTACATAAAA 660

pUSA05-1-SUR11 ATGAAATGCGATTAAGCTTTTTCTAATTCACATAAGCGTGCAGGTTTAAAGTACATAAAA 660

pUSA03 ATGAAATGCGATTAAGCTTTTTCTAATTCACATAAGCGTGCAGGTTTAAAGTACATAAAA 660

SG70 ATGAAATGCGATTAAGCTTTTTCTAATTCACATAAGCGTGCAGGTTTAAAGTACATAAAA 660

************************************************************

M260_MSHR AATATAATGAAAAAAAGCATCATTATACTAACGTTATACCAACATTATACTCTCATTATA 720

pUSA05-1-SUR11 AATATAATGAAAAAAAGCATCATTATACTAACGTTATACCAACATTATACTCTCATTATA 720

pUSA03 AATATAATGAAAAAAAGCATCATTATACTAACGTTATACCAACATTATACTCTCATTATA 720

SG70 AATATAATGAAAAAAAGCATCATTATACTAACGTTATACCAACATTATACTCTCATTATA 720

************************************************************

M260_MSHR CTAATTGCTTATTCCAATTTCCTATTGGTTGGAACCAACAGGCGTTAGTGTGTTGTTGAG 780

pUSA05-1-SUR11 CTAATTGCTTATTCCAATTTCCTATTGGTTGGAACCAACAGGCGTTAGTGTGTTGTTGAG 780

pUSA03 CTAATTGCTTATTCCAATTTCCTATTGGTTGGAACCAACAGGCGTTAGTGTGTTGTTGAG 780

SG70 CTAATTGCTTATTCCAATTTCCTATTGGTTGGAACCAACAGGCGTTAGTGTGTTGTTGAG 780

************************************************************

M260_MSHR TTGGTACTTTCATGGGATTAATCCCATGAAACCCCCAACCAACTCGCCAAAGCTTTGGCT 840

pUSA05-1-SUR11 TTGGTACTTTCATGGGATTAATCCCATGAAACCCCCAACCAACTCGCCAAAGCTTTGGCT 840

pUSA03 TTGGTACTTTCATGGGATTAATCCCATGAAACCCCCAACCAACTCGCCAAAGCTTTGGCT 840

SG70 TTGGTACTTTCATGGGATTAATCCCATGAAACCCCCAACCAACTCGCCAAAGCTTTGGCT 840

************************************************************

M260_MSHR AACACACACGCCATTCCAACCAATAGTTTTCTCGGCATAAAGCCATGCTCTGACGCTTAA 900

pUSA05-1-SUR11 AACACACACGCCATTCCAACCAATAGTTTTCTCGGCATAAAGCCATGCTCTGACGCTTAA 900

pUSA03 AACACACACGCCATTCCAACCAATAGTTTTCTCGGCATAAAGCCATGCTCTGACGCTTAA 900

SG70 AACACACACGCCATTCCAACCAATAGTTTTCTCGGCATAAAGCCATGCTCTGACGCTTAA 900

************************************************************

M260_MSHR ATGCACTAATGCCTTAAAAAAACATTAAAGTCTAAC----ACACTAGACTTATTTACTTC 956

pUSA05-1-SUR11 ATGCACTAATGCCTTAAAAAAACATTAAAGTCTAAC----ACACTAGACTTATTTACTTC 956

pUSA03 ATGCACTAATGCCTTAAAAAAACATTAAAGTCTAAC----ACACTAGACTTATTTACTTC 956

SG70 ATGCACTAATGCCTTAAAAAAACATTAAAGTCTAACTAACACACTAGACTTATTTACTTC 960

************************************ ********************

M260_MSHR GTAATTAAGTCGTTAAACCGTGTGCTCTACGACCAAAAGTATAAAACCTTTAAGAACTTT 1016

pUSA05-1-SUR11 GTAATTAAGTCGTTAAACCGTGTGCTCTACGACCAAAAGTATAAAACCTTTAAGAACTTT 1016

pUSA03 GTAATTAAGTCGTTAAACCGTGTGCTCTACGACCAAAAGTATAAAACCTTTAAGAACTTT 1016

SG70 GTAATTAAGTCGTTAAACCGTGTGCTCTACGACCAAAAGTATAAAACCTTTAAGAACTTT 1020

************************************************************

M260_MSHR CTTTTTTCTTGTAAAAAAAGAAACTAGATAAATCTCTCATATCTTTTATTCAATAATCGC 1076

pUSA05-1-SUR11 CTTTTTTCTTGTAAAAAAAGAAACTAGATAAATCTCTCATATCTTTTATTCAATAATCGC 1076

pUSA03 CTTTTTTCTTGTAAAAAAAGAAACTAGATAAATCTCTCATATCTTTTATTCAATAATCGC 1076

SG70 CTTTTTTCTTGTAAAAAAAGAAACTAGATAAATCTCTCATATCTTTTATTCAATAATCGC 1080

************************************************************

M260_MSHR ATCAGATTGCAGTATAAATTTAACGATCACTCATCATGTTCATATTTATCAGAGCTCGTG 1136

pUSA05-1-SUR11 ATCAGATTGCAGTATAAATTTAACGATCACTCATCATGTTCATATTTATCAGAGCTCGTG 1136

pUSA03 ATCAGATTGCAGTATAAATTTAACGATCACTCATCATGTTCATATTTATCAGAGCTCGTG 1136

SG70 ATCAGATTGCAGTATAAATTTAACGATCACTCATCATGTTCATATTTATCAGAGCTCGTG 1140

************************************************************

M260_MSHR CTATAATTATACTAATTTTATAAGGAGGAAAAAATATGGGCATTTTTAGTATTTTTGTAA 1196

pUSA05-1-SUR11 CTATAATTATACTAATTTTATAAGGAGGAAAAAATATGGGCATTTTTAGTATTTTTGTAA 1196

pUSA03 CTATAATTATACTAATTTTATAAGGAGGAAAAAATA------------------------ 1172

SG70 CTATAATTATACTAATTTTATAAGGAGGAAAAAATA------------------------ 1176

************************************

**IR1 IR2**

M260_MSHR TCAGCACAGTTCATTATCAACCAAACAAAAAATAAGTGGTTATAATGAATCGTTAATAAG 1256

pUSA05-1-SUR11 TCAGCACAGTTCATTATCAACCAAACAAAAAATAAGTGGTTATAATGAATCGTTAATAAG 1256

pUSA03 ------------------------------------------------------------

SG70 ------------------------------------------------------------

**IR3 IR4**

M260_MSHR CAAAATTCATTATAACCAAATTAAAGAGGGTTATAATGAACGAGAAAAATATAAAACACA 1316

pUSA05-1-SUR11 CAAAATTCATTATAACCAAATTAAAGAGGGTTATAATGAACGAGAAAAATATAAAACACA 1316

pUSA03 -----------------------AAGAGGGTTATAATGAACGAGAAAAATATAAAACACA 1209

SG70 -----------------------AAGAGGGTTATAATGAACGAGAAAAATATAAAACACA 1213

*************************************

|→ ***erm(C)***

M260_MSHR GTCAAAACTTTATTACTTCAAAACATAATATAGATAAAATAATGACAAATATAAGATTAA 1376

pUSA05-1-SUR11 GTCAAAACTTTATTACTTCAAAACATAATATAGATAAAATAATGACAAATATAAGATTAA 1376

pUSA03 GTCAAAACTTTATTACTTCAAAACATAATATAGATAAAATAATGACAAATATAAGATTAA 1269

SG70 GTCAAAACTTTATTACTTCAAAACATAATATAGATAAAATAATGACAAATATAAGATTAA 1273

************************************************************

M260_MSHR ATGAACATGATAATATCTTTGAAATCGGCTCAGGAAAAGGGCATTTTACCCTTGAATTAG 1436

pUSA05-1-SUR11 ATGAACATGATAATATCTTTGAAATCGGCTCAGGAAAAGGGCATTTTACCCTTGAATTAG 1436

pUSA03 ATGAACATGATAATATCTTTGAAATCGGCTCAGGAAAAGGGCATTTTACCCTTGAATTAG 1329

SG70 ATGAACATGATAATATCTTTGAAATCGGCTCAGGAAAAGGGCATTTTACCCTTGAATTAG 1333

************************************************************

M260_MSHR TACAGAGGTGTAATTTCGTAACTGCCATTGAAATAGACCATAAATTATGCAAAACTACAG 1496

pUSA05-1-SUR11 TACAGAGGTGTAATTTCGTAACTGCCATTGAAATAGACCATAAATTATGCAAAACTACAG 1496

pUSA03 TACAGAGGTGTAATTTCGTAACTGCCATTGAAATAGACCATAAATTATGCAAAACTACAG 1389

SG70 TACAGAGGTGTAATTTCGTAACTGCCATTGAAATAGACCATAAATTATGCAAAACTACAG 1393

************************************************************

M260_MSHR AAAATAAACTTGTTGATCACGATAATTTCCAAGTTTTAAACAAGGATATATTGCAGTTTA 1556

pUSA05-1-SUR11 AAAATAAACTTGTTGATCACGATAATTTCCAAGTTTTAAACAAGGATATATTGCAGTTTA 1556

pUSA03 AAAATAAACTTGTTGATCACGATAATTTCCAAGTTTTAAACAAGGATATATTGCAGTTTA 1449

SG70 AAAATAAACTTGTTGATCACGATAATTTCCAAGTTTTAAACAAGGATATATTGCAGTTTA 1453

************************************************************

M260_MSHR AATTTCCTAAAAACCAATCCTATAAAATATTTGGTAATATACCTTATAACATAAGTACGG 1616

pUSA05-1-SUR11 AATTTCCTAAAAACCAATCCTATAAAATATTTGGTAATATACCTTATAACATAAGTACGG 1616

pUSA03 AATTTCCTAAAAACCAATCCTATAAAATATTTGGTAATATACCTTATAACATAAGTACGG 1509

SG70 AATTTCCTAAAAACCAATCCTATAAAATATTTGGTAATATACCTTATAACATAAGTACGG 1513

************************************************************

M260_MSHR ATATAATACGCAAAATTGTTTTTGATAGTATAGCTGATGAGATTTATTTAATCGTGGAAT 1676

pUSA05-1-SUR11 ATATAATACGCAAAATTGTTTTTGATAGTATAGCTGATGAGATTTATTTAATCGTGGAAT 1676

pUSA03 ATATAATACGCAAAATTGTTTTTGATAGTATAGCTGATGAGATTTATTTAATCGTGGAAT 1569

SG70 ATATAATACGCAAAATTGTTTTTGATAGTATAGCTGATGAGATTTATTTAATCGTGGAAT 1573

************************************************************

M260_MSHR ACGGGTTTGCTAAAAGATTATTAAATACAAAACGCTCATTGGCATTATTTTTAATGGCAG 1736

pUSA05-1-SUR11 ACGGGTTTGCTAAAAGATTATTAAATACAAAACGCTCATTGGCATTATTTTTAATGGCAG 1736

pUSA03 ACGAGTTTGCTAAAAGATTATTAAATACAAAACGCTCATTGGCATTATTTTTAATGGCAG 1629

SG70 ACGAGTTTGCTAAAAGATTATTAAATACAAAACGCTCATTGGCATTATTTTTAATGGCAG 1633

*** ********************************************************

M260_MSHR AAGTTGATATTTCTATATTAAGTATGGTTCCAAGAGAATATTTTCATCCTAAACCTAAAG 1796

pUSA05-1-SUR11 AAGTTGATATTTCTATATTAAGTATGGTTCCAAGAGAATATTTTCATCCTAAACCTAAAG 1796

pUSA03 AAGTTGATATTTCTATATTAAGTATGGTTCCAAGAGAATATTTTCATCCTAAACCTAAAG 1689

SG70 AAGTTGATATTTCTATATTAAGTATGGTTCCAAGAGAATATTTTCATCCTAAACCTAAAG 1693

************************************************************

M260_MSHR TGAATAGCTCACTTATCAGATTAAATAGAAAAAAATCAAGAATATCACACAAAGATAAAC 1856

pUSA05-1-SUR11 TGAATAGCTCACTTATCAGATTAAATAGAAAAAAATCAAGAATATCACACAAAGATAAAC 1856

pUSA03 TGAATAGCTCACTTATCAGATTAAATAGAAAAAAATCAAGAATATCACACAAAGATAAAC 1749

SG70 TGAATAGCTCACTTATCAGATTAAATAGAAAAAAATCAAGAATATCACACAAAGATAAAC 1753

************************************************************

M260_MSHR AGAAGTATAATTATTTCGTTATGAAATGGGTTAACAAAGAATACAAGAAAATATTTACAA 1916

pUSA05-1-SUR11 AGAAGTATAATTATTTCGTTATGAAATGGGTTAACAAAGAATACAAGAAAATATTTACAA 1916

pUSA03 AGAAGTATAATTATTTCGTTATGAAATGGGTTAACAAAGAATACAAGAAAATATTTACAA 1809

SG70 AGAAGTATAATTATTTCGTTATGAAATGGGTTAACAAAGAATACAAGAAAATATTTACAA 1813

************************************************************

M260_MSHR AAAATCAATTTAACAATTCCTTAAAACATGCAGGAATTGACGATTTAAACAATATTAGCT 1976

pUSA05-1-SUR11 AAAATCAATTTAACAATTCCTTAAAACATGCAGGAATTGACGATTTAAACAATATTAGCT 1976

pUSA03 AAAATCAATTTAACAATTCCTTAAAACATGCAGGAATTGACGATTTAAACAATATTAGCT 1869

SG70 AAAATCAATTTAACAATTCCTTAAAACATGCAGGAATTGACGATTTAAACAATATTAGCT 1873

************************************************************

M260_MSHR TTGAACAATTCTTATCTCTTTTCAATAGCTATAAATTATTTAATAAGTAA 2026

pUSA05-1-SUR11 TTGAACAATTCTTATCTCTTTTCAATAGCTATAAATTATTTAATAAGTAA 2026

pUSA03 TTGAACAATTCTTATCTCTTTTCAATAGCTATAAATTATTTAATAAGTAA 1919

SG70 TTGAACAATTCTTATCTCTTTTCAATAGCTATAAATTATTTAATAAGTAA 1923

**************************************************

**Figure S2** Alignment of *repL*, *erm(C)* and its promoter region of *S. aureus* strain USA300_FPR3757 (plasmid pSA03) and USA300-SUR11 (plasmid pUSA05-1-SUR11), *S. argenteus* strain SaM260_MSHR and SG70 (present study). Start and termination codons of *repL*/*erm(C)* are shown in yellow and blue, respectively. Asterisk denotes identical amino acid. Start codon and termination codon of leader peptide (19 amino acids) in pUSA05-1-SUR11 and M260_MSHR are shown by boxes with shade. Inverted repeats (IR1-IR4) are indicated by arrows, and ribosomal binding sites are underlined.
