# Supplementary material for: Distribution of Virulence Factors and Resistance Determinants in Three Genotypes of Staphylococcus argenteus Clinical Isolates in Japan
Source: Pathogens. 2021 Feb 3;10(2):163. doi: 10.3390/pathogens10020163 (PMC7913748; doi:10.3390/pathogens10020163)
Supplement: Supplementary file 1 [file pathogens-10-00163-s001.zip › Suppl-20210129/Figure-S3.docx]

GyrA

▮▮ ▮

SG103 DKSYKKSARIVGDVMGKYHPHGDSSIYEAMVRMAQDFSYRYPLVDGQGNFGSMDGDGAAA 120

SG95 DKSYKKSARIVGDVMGKYHPHGDSSIYEAMVRMAQDFSYRYPLVDGQGNFGSMDGDGAAA 120

MSHR1132 DKSYKKSARIVGDVMGKYHPHGDSSIYEAMVRMAQDFSYRYPLVDGQGNFGSMDGDGAAA 120

XNO106 DKSYKKSARIVGDVMGKYHPHGDSSIYEAMVRMAQDFSYRYPLVDGQGNFGSMDGDGAAA 120

SG99 DKSYKKSARIVGDVMGKYHPHGDSSIYEAMVRMAQDFSYRYPLVDGQGNFGSMDGDGAAA 120

N315 DKSYKKSARIVGDVMGKYHPHGDSSIYEAMVRMAQDFSYRYPLVDGQGNFGSMDGDGAAA 120

NCTC8325 DKSYKKSARIVGDVMGKYHPHGDSSIYEAMVRMAQDFSYRYPLVDGQGNFGSMDGDGAAA 120

************************************************************

GyrB

▮ ▮ ▮

SG103 CSSKSPEECEIFLVEGDSAGGSTKSGRDSRTQAILPLRGKILNVEKARLDRILNNNEIRQ 480

SG95 CSSKSPEECEIFLVEGDSAGGSTKSGRDSRTQAILPLRGKILNVEKARLDRILNNNEIRQ 480

XNO106 CSSKSPEECEIFLVEGDSAGGSTKSGRDSRTQAILPLRGKILNVEKARLDRILNNNEIRQ 480

SG99 CSSKSPEECEIFLVEGDSAGGSTKSGRDSRTQAILPLRGKILNVEKARLDRILNNNEIRQ 480

MSHR1132 CSSKSPEECEIFLVEGDSAGGSTKSGRDSRTQAILPLRGKILNVEKARLDRILNNNEIRQ 480

N315 CSSKSPEECEIFLVEGDSAGGSTKSGRDSRTQAILPLRGKILNVEKARLDRILNNNEIRQ 480

NCTC8325 CSSKSPEECEIFLVEGDSAGGSTKSGRDSRTQAILPLRGKILNVEKARLDRILNNNEIRQ 480

************************************************************

ParC

▮▮ ▮

N315 RKSAKTVGDVIGQYHPHGDSSVYEAMVRLSQDWKLRHVLIEMHGNNGSIDNDPPAAMRYT 120

NCTC8325 RKSAKTVGDVIGQYHPHGDSSVYEAMVRLSQDWKLRHVLIEMHGNNGSIDNDPPAAMRYT 120

XNO106 RKSAKTVGDVIGQYHPHGDSSVYEAMVRLSQDWKLRHVLIEMHGNNGSIDNDPPAAMRYT 120

SG103 RKSAKTVGDVIGQYHPHGDSSVYEAMVRLSQDWKLRHVLIEMHGNNGSIDNDPPAAMRYT 120

SG95 RKSAKTVGDVIGQYHPHGDSSVYEAMVRLSQDWKLRHVLIEMHGNNGSIDNDPPAAMRYT 120

SG99 RKSAKTVGDVIGQYHPHGDSSVYEAMVRLSQDWKLRHVLIEMHGNNGSIDNDPPAAMRYT 120

MSHR1132 RKSAKTVGDVIGQYHPHGDSSVYEAMVRLSQDWKLRHVLIEMHGNNGSIDNDPPAAMRYT 120

************************************************************

ParE

▮ ▮

SG103 KNTEKNELYLVEGDSAGGSAKLGRDRKFQAILPLRGKVINTEKARLEDIFKNEEINTIIH 478

SG95 KNTEKNELYLVEGDSAGGSAKLGRDRKFQAILPLRGKVINTEKARLEDIFKNEEINTIIH 478

SG99 KNTEKNELYLVEGDSAGGSAKLGRDRKFQAILPLRGKVINTEKARLEDIFKNEEINTIIH 478

XNO106 KNTEKNELYLVEGDSAGGSAKLGRDRKFQAILPLRGKVINTEKARLEDIFKNEEINTIIH 478

MSHR1132 KNTEKNELYLVEGDSAGGSAKLGRDRKFQAILPLRGKIINTEKARLEDIFKNEEINTIIH 478

N315 KNTEKNELYLVEGDSAGGSAKLGRDRKFQAILPLRGKVINTEKARLEDIFKNEEINTIIH 480

NCTC8325 KNTEKNELYLVEGDSAGGSAKLGRDRKFQAILPLRGKVINTEKARLEDIFKNEEINTIIH 478

*************************************:**********************

**Figure S3.** Alignment of partial GyrA GyrB, ParC, and ParE amino acid sequences including quinolone-resistance determining regions (QRDR) of *S. argenteus* strains (SG95, SG99, SG103, XNO106, and MSHR1132) and *S. aureus* strains (N315 and NCTC8325) that are fluoroquinolone-susceptible (This study, [59]). Asterisk below the alignment indicates identical amino acids. Vertical bars indicate positions where substitution of amino acid was reported in fluoroquinolone-resistant strains [57-59].
