# Supplementary material for: Distribution of Virulence Factors and Resistance Determinants in Three Genotypes of Staphylococcus argenteus Clinical Isolates in Japan
Source: Pathogens. 2021 Feb 3;10(2):163. doi: 10.3390/pathogens10020163 (PMC7913748; doi:10.3390/pathogens10020163)
Supplement: Supplementary file 1 [file pathogens-10-00163-s001.zip › Suppl-20210129/TableS2-R2ü@.docx]

**Table S2 Drug resistance profile and prevalence of virulence factors/drug resistance genes in 82 *S. argenteus* isolates in Hokkaido, Japan for one year-period (August 2019 - July 2020)**
