# Supplementary material for: Distribution of Virulence Factors and Resistance Determinants in Three Genotypes of Staphylococcus argenteus Clinical Isolates in Japan
Source: Pathogens. 2021 Feb 3;10(2):163. doi: 10.3390/pathogens10020163 (PMC7913748; doi:10.3390/pathogens10020163)
Supplement: Supplementary file 1 [file pathogens-10-00163-s001.zip › Suppl-20210129/TableS3-R.docx]

**Table S3 Sequence identity of component genes *(seg-sei-sem-sen-seo-seu*) in *egc-2* of *S. argenteus* SG48 in the present study to those of *S. argenteus*, *S. aureus* and *S. schweitzeri* isolates reported previously**
