# Supplementary material for: Distribution of Virulence Factors and Resistance Determinants in Three Genotypes of Staphylococcus argenteus Clinical Isolates in Japan
Source: Pathogens. 2021 Feb 3;10(2):163. doi: 10.3390/pathogens10020163 (PMC7913748; doi:10.3390/pathogens10020163)
Supplement: Supplementary file 1 [file pathogens-10-00163-s001.zip › Suppl-20210129/TableS4-R.docx]

**Table S4 Nucleotide sequence identity of drug resistance genes detected in the present study to those of *S. aureus* and staphylococcal species and enterococcus retrieved from GenBank database**
