# Supplementary material for: Distribution of Virulence Factors and Resistance Determinants in Three Genotypes of Staphylococcus argenteus Clinical Isolates in Japan
Source: Pathogens. 2021 Feb 3;10(2):163. doi: 10.3390/pathogens10020163 (PMC7913748; doi:10.3390/pathogens10020163)
Supplement: Supplementary file 1 [file pathogens-10-00163-s001.zip › Suppl-20210129/TableS6-R.docx]

**Table S6 GenBank accession numbers assigned to *lukS-PV-lukF-PV*, *sea*, *seb*, *sec*, *egc-2*, *tst-1*, *blaZ*, SCC*mec* complex, *erm(C)*, *msr(A)*, *lnuA*, *tet(K)*, *aac(6')-Ie-aph(2'')-Ia*, *gyrA/B*, *parC/E* genes detected in the present study**
